# Identification of RP11‐770J1.4 as immune‐related lncRNA regulating the CTXN1–cGAS–STING axis in histologically lower‐grade glioma

**DOI:** 10.1002/mco2.458

**Published:** 2023-12-19

**Authors:** Qiyuan Zhuang, Chaxian Liu, Yihan Hu, Ying Liu, Yingying Lyu, Yuheng Liao, Liang Chen, Hui Yang, Ying Mao

**Affiliations:** ^1^ Department of Neurosurgery Huashan Hospital, Fudan University Shanghai China; ^2^ School of Life Sciences, Fudan University Shanghai China; ^3^ Department of Pathology School of Basic Medical Sciences, Fudan University Shanghai China; ^4^ Key Laboratory of Medical Epigenetics and Metabolism and Molecular and Cell Biology Lab Institute of Biomedical Sciences, Shanghai Medical College, Fudan University Shanghai China; ^5^ National Center for Neurological Disorders Huashan Hospital, Fudan University Shanghai China; ^6^ Institute for Translational Brain Research Shanghai Medical College, Fudan University Shanghai China; ^7^ State Key Laboratory of Medical Neurobiology and MOE Frontiers Center for Brain Science, Institutes of Brain Science, Fudan University Shanghai China

**Keywords:** ceRNA network, cytosolic DNA sensing pathway, immune‐related lncRNA, lower‐grade glioma, single‐cell RNA sequencing

## Abstract

Human gliomas are lethal brain cancers. Emerging evidence revealed the regulatory role of long noncoding RNAs (lncRNAs) in tumors. Here, we performed a comprehensive analysis of the expression profiles of RNAs in histologically lower‐grade glioma (LGG). Enrichment analysis revealed that glioma is influenced by immune‐related signatures. Survival analysis further established the close correlation between network features and glioma prognosis. Subsequent experiments showed lncRNA RP11‐770J1.4 regulates *CTXN1* expression through hsa‐miR‐124‐3p. Correlation analysis identified lncRNA RP11‐770J1.4 was immune related, specifically involved in the cytosolic DNA sensing pathway. Downregulated lncRNA RP11‐770J1.4 resulted in increased spontaneous gene expression of the cGAS–STING pathway. Single‐cell RNA sequencing analysis, along with investigations in a glioblastoma stem cell model and patient sample analysis, demonstrated the predominant localization of *CTXN1* within tumor cores rather than peripheral regions. Immunohistochemistry staining established a negative correlation between CTXN1 expression and infiltration of CD8^+^ T cells. In vivo, *Ctxn1* knockdown in GL261 cells led to decreased tumor burden and improved survival while increasing infiltration of CD8^+^ T cells. These findings unveil novel insights into the lncRNA RP11‐770J1.4–CTXN1 as a potential immune regulatory axis, highlighting its therapeutic implications for histologically LGGs.

## Graphical abstract

A comprehensive analysis of the expression profiles of RNAs in histologically lower‐grade glioma (LGG) was performed to reveal the influence of immune‐related signatures on glioma biology. Survival analysis established the close correlation between network features and glioma prognosis. Of which, RP11‐770J1.4 as a regulator of CTXN1 expression by sponging hsa‐miR‐124‐3p. Downregulated RP11‐770J1.4 resulted in increased spontaneous gene expression of the cGAS–STING pathway. *Ctxn1* knockdown in GL261 cells led to decreased tumor weight and prolonged survival time while increasing tumor CD8^+^ T cells infiltration. These findings uncover the RP11‐770J1.4–CTXN1 as a novel immune regulatory axis in gliomas.

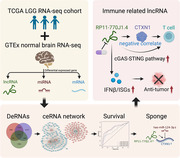

## 1 INTRODUCTION

Glioma is the most prevalent and lethal type of primary malignant tumor within the brain.^1^, ^2^ Despite advanced strides in multimodal therapeutic approaches, encompassing surgical resection, radiotherapy, and chemotherapy,^3^ the median overall survival (OS) of glioblastoma multiforme remains less than 15 months.^4^, ^5^, ^6^, ^7^ Lower‐grade glioma (LGG), corresponding to WHO grades II and III, generally has a more favorable prognosis compared with GBM.^8^ However, not all histologically LGG has a relatively favorable prognosis. Most diffuse grade II and anaplastic grade III IDH‐wild‐type astrocytomas exhibit a high degree of malignancy and a similar outcome to grade IV GBM.^9^, ^10^, ^11^ Therefore, there is an urgent need for novel molecular biomarkers of glioma.

Noncoding RNAs (ncRNAs) have assumed a pivotal role as regulators of gene expression across diverse biological processes and disease contexts.^12^, ^13^ Within the spectrum of ncRNAs, which includes circular RNAs and long noncoding RNAs (lncRNAs),^14^, ^15^, ^16^ these molecules commonly exhibit dysregulation in various tumor types.^17^, ^18^ Pan‐cancer transcriptome analysis highlights diverse lncRNAs as potential prognostic biomarkers across different cancer types.^19^, ^20^ LncRNAs can act as competing endogenous RNAs (ceRNAs) for miRNAs,^21^ modulating the expression of mRNAs. Moreover, recent studies have also demonstrated that lncRNAs could influence the tumor microenvironment and immune system.^22^, ^23^, ^24^, ^25^ For example, the lncRNA *NRON* has been shown to promote the maintenance of the resting state of T cells by inhibiting the nuclear translocation of phosphorylated NFAT.^26^ Tumor‐specific expression of lncRNA *LINK‐A* in mouse mammillary glands induces tumors that resemble human triple‐negative breast cancer (TNBC).^25^ The levels of *LINK‐A* are elevated in patients with PD‐1 blockade‐resistant TNBC. Thus, further investigation of immune‐related lncRNAs may provide novel therapeutic targets for cancer treatment.

Conventional RNA sequencing methods only provide average gene expression levels for all cells in a tumor, which masks the variability between cells within a tumor. Single‐cell sequencing is a new technology that acquires the genome, transcriptome, and epigenome of a cell from a single‐cell dimension and performs high‐throughput sequencing.^27^ This technology can reveal the gene structural and expression status of individual cells, reflecting the heterogeneity among cells, and is a powerful means to study complex cell populations and to analyze individual cells with multiple parameters.^28^ Using single‐cell genomic analysis, Richard et al.^29^ identified different states of cancer stem cells and their differentiated progeny in gliomas. These cellular states are strongly associated with high and low glioma grades and have the potential for tumor plasticity. Yeo et al.^30^ revealed longitudinal holistic changes in immune cell composition throughout the tumor progression using flow cytometry coupled with single‐cell RNA sequencing (scRNA‐seq). Thus, single‐cell sequencing is a compelling approach to studying gene expression in tumor cells in relation to other cell populations.

The immune microenvironment of gliomas is an important factor influencing tumor prognosis. Immunotherapy, which involves modulating the function of the immune system through stimulatory or inhibitory mechanisms mediated by immune checkpoint molecules, has been used with great success in solid tumors.^31^ However, clinical trials targeting immune checkpoints have not shown improved survival benefits in GBM patients.^32^, ^33^, ^34^ Blockade of negative feedback signals from immune checkpoints can provoke an immune response in the body, which in turn promotes T cell activity to kill tumors. However, the use of immune checkpoint inhibitors alone cannot reverse the immune‐depleting effects of GBM on T cells. Therefore, it is important to explore what tumor cell genes are associated with infiltrating immune cell infiltration.

In this study, we performed a comprehensive transcriptome analysis of The Cancer Genome Atlas (TCGA)‐LGG (511 samples) and compared it with that of normal brain tissues (209 samples) from the Genotype‐Tissue Expression (GTEx) project. We identified 703 mRNAs, 1626 lncRNAs, and 90 miRNAs that were differentially expressed in LGG. Based on the predicted target miRNAs and mRNAs, we constructed a ceRNA network comprising 316 RNA pairs characterized by negative regulatory interactions. Survival analyses identified several prognostic biomarkers for histologically LGGs. Among these genes, *CTXN1*, which is involved in neuronal signaling and cortical development, was overexpressed in gliomas with poor prognosis. Subsequent validation confirmed that lncRNA RP11‐770J1.4 regulate *CTXN1* in vitro through hsa‐miR‐124‐3p. Additionally, lncRNA RP11‐770J1.4 displayed significant associations with immune‐related factors, and its suppression amplified the activation of cytosolic DNA sensing pathways. We also examined *CTXN1* expression by patient samples, the glioblastoma stem cell (GSC) model (MES28) and scRNA‐seq (3505 cells), finding a negative correlation between *CTXN1* and CD8^+^ T cell infiltration, as evidenced by Immunohistochemistry staining. Knockdown of *Ctxn1* not only curtailed tumor growth but also improved survival in vivo, concomitant with an augmentation in CD8^+^ T‐cell infiltration. Hence, our ceRNA network unveils novel insights into the lncRNA RP11‐770J1.4–CTXN1 as a potential immune regulatory axis, suggesting its therapeutic implications for histologically LGGs.

## 2 RESULTS

### 2.1 Differentially expressed RNAs identification in histologically LGG

**FIGURE 1:**
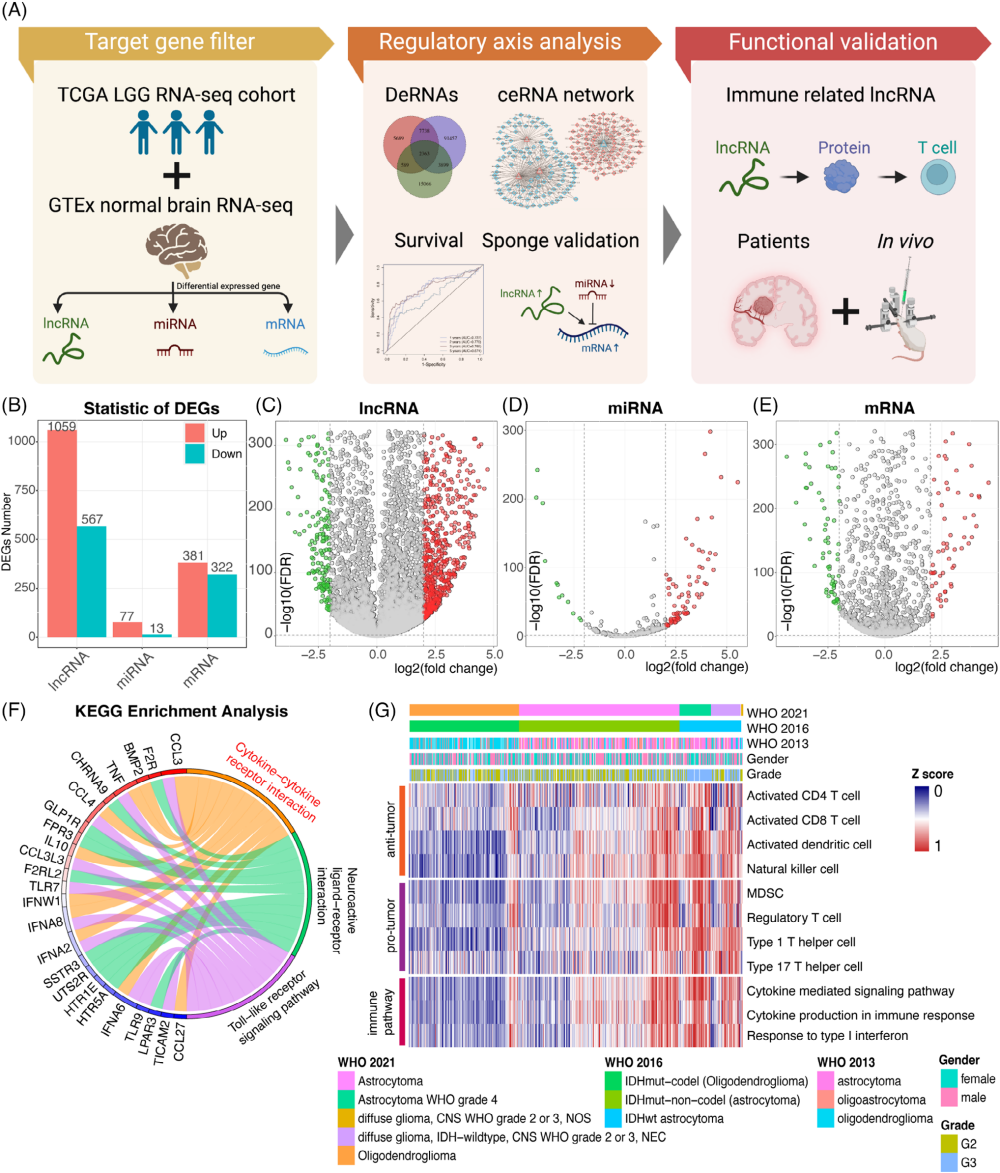
Differential expression and functional analysis of lncRNAs, miRNAs, and mRNAs in glioma. (A) Schematic representation of the study design. (B) Bar chart illustrating the total count of DEG genes for lncRNAs, miRNAs and mRNAs. (C–E) Differential expression of lncRNAs, miRNAs, and mRNAs represented by volcano plots. Each data point corresponds to a gene, with green indicating downregulated DEGs, red indicating upregulated DEGs, and grey indicating non‐DEGs. Dashed vertical lines denote a threshold of 4‐fold change (log_2_FC), while the horizontal dashed line represents a false discovery rate (FDR) < 0.05. (F) Circular plot depicting enriched Kyoto Encyclopedia of Genes and Genomes (KEGG) pathways. Functions or pathways are depicted on the right half, and associated genes on the left half. Gene colors vary from red to blue, indicating the log_2_FC magnitude. (G) Heatmap displaying ssGSEA scores for marker gene lists related to immune cells or pathways. *Z* scores are normalized relative to all cases, with clinical characteristics annotated in column labels.

We analyzed the differences between tumor and normal brains at the different RNA levels to gain multiple perspectives on LGG. The study design is shown in the flow chart (Figure 1A). RNA expressions were retrieved from a total of 209 normal brain tissues (GTEx) and 511 TCGA‐LGG samples. Subsequently, edgeR package was used to normalize read counts for all genes. A total of 1626 differentially expressed lncRNAs (DElncRNAs), 90 differentially expressed miRNAs (DEmiRNAs), and 703 differentially expressed mRNAs (DEmRNAs) were identified (Figure 1B). We visualized statistically significant DElncRNAs, DEmiRNAs, and DEmRNAs through volcano plots (Figures 1C–E). Concurrently, complete clustering based on Euclidean distance was applied to the three groups (Figures S1A–C).

### 2.2 IDH‐wildtype histologically LGG enriched higher immune‐related signatures

We then conducted Gene Ontology (GO) enrichment and Kyoto Encyclopedia of Genes and Genomes (KEEG) pathway analyses to elucidate the putative functions of the 703 identified mRNAs. Top hits terms were enriched, such as “chemokine‐mediated signaling pathway” and “inflammatory response.” It indicated that immune responses were related to gliomas. GO terms were divided into three major branches and the five most significant functions were plotted in circular form (Figure S1D). We also visualized the degree of enrichment of each GO term and pathway (Figure S1E). Based on KEGG pathway analysis, several immune pathways were identified, such as “Toll‐like receptor signaling pathway” and “cytokine–cytokine receptor interaction” (Figure 1F). Representative gene lists of DEmRNA‐derived immune pathways and pro‐tumor or anti‐tumor immune cells were analyzed in the LGG cohort (Figure 1G). We found that, compared with IDH‐mutant histologically LGGs, IDH‐wildtype histologically LGGs enriched stronger immune‐related signatures. Thus, further investigation is needed to reveal how the immune response contributes to histologically LGGs.

### 2.3 CeRNA network of histologically LGG showed potential regulatory axes

**FIGURE 2:**
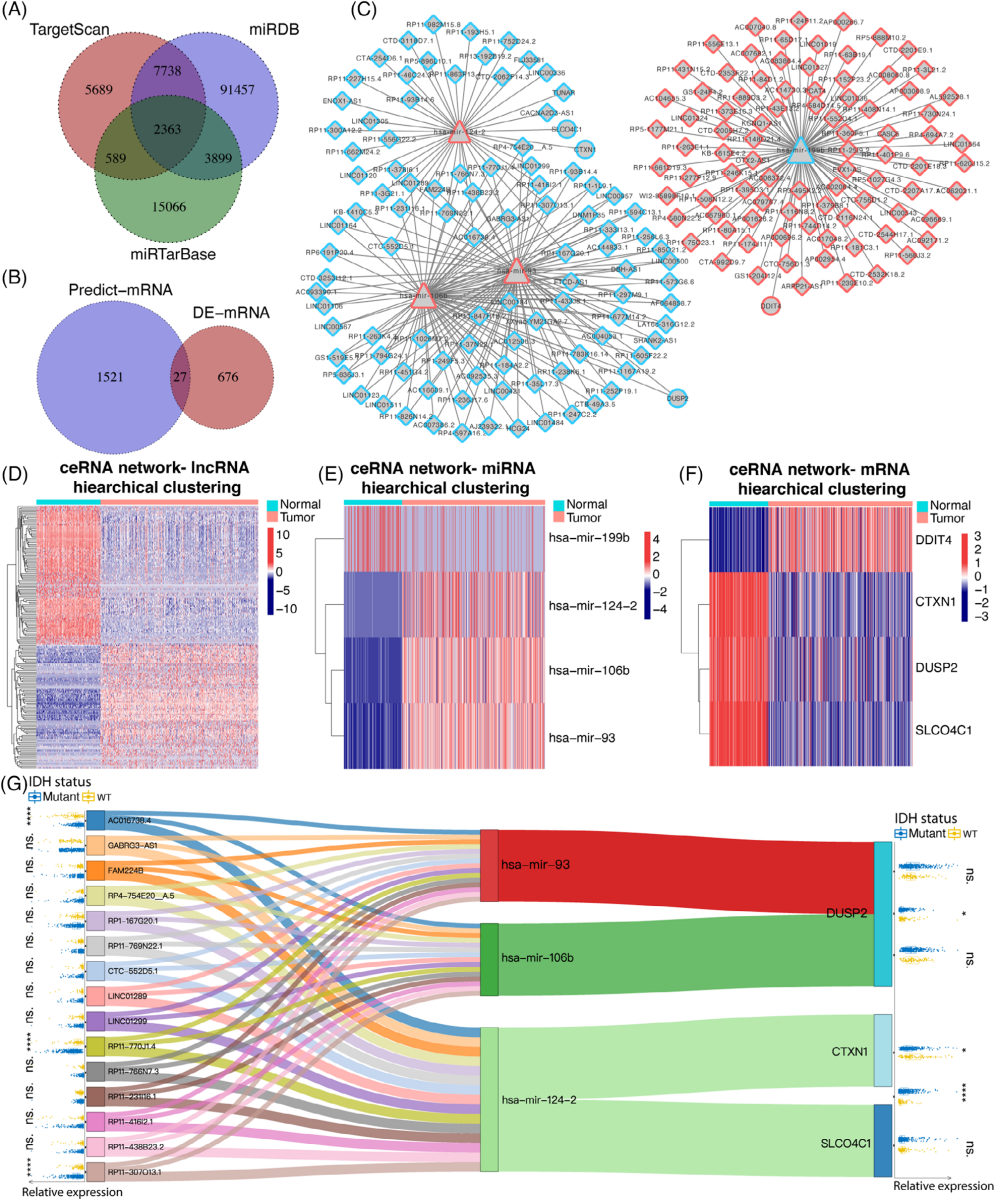
Construction and characterization of a regulatory network in glioma. (A) The process of DEmiRNAs was illustrated by Venn diagram. (B) Venn diagram depicting predicted target mRNAs of DEmiRNAs. (C) Schematic representation of the regulatory ceRNA network in histologically LGG. Red indicates upregulation, while blue indicates downregulation. Diamonds represent DElncRNAs, triangles represent DEmiRNAs and circles represent DEmRNAs. (D–F) Heatmap plots illustrating DElncRNAs in the left panel, DEmiRNAs in the middle panel, and DEmRNAs in the right panel of ceRNA network. The color gradient represents the log_2_FC of gene expression. (G) Sankey plot of regulatory axes (DElncRNA–DEmiRNA–DEmRNA). The top 15 lncRNAs in the ceRNA network were ranked by node degree and visualized. The relative expression of each gene within the ceRNA network between IDH‐mutant and IDH‐wildtype groups is depicted in the left or right field.

Subsequently, targeted miRNAs of DElncRNAs were predicted by miRcode software. The intersection of DElncRNAs with DEmiRNAs resulted in the identification of 1970 relationship pairs (Table S1). In parallel, we predicted the target mRNAs of the DEmiRNAs using three software programs: TargetScan, miRDB, and miRTarBase. The DEmiRNA–mRNA intersection pairs obtained from these three programs yielded 2363 relationships in total (Figure 2A; Table S2). Among these pairs, we mapped a total of 27 DEmRNAs (Figure 2B; Table S3). We following identified 6847 ceRNA triads (DElncRNA–DEmiRNA–DEmRNA). Among these, 316 ceRNA pairs had negative regulatory relationships, indicating that the lncRNA and mRNA are regulated in the same direction, opposite that of the miRNA. The ceRNA network was constructed which compromised 282 edges and 195 nodes (Figure 2C). Additionally, we generated heatmaps of lncRNAs, miRNAs, and mRNAs from the ceRNA network (Figures 2D–F). We then calculated the degree of each gene's network node based on the network topology. A lncRNA–miRNA–mRNA negative regulation axis was constructed using the 15 top lncRNAs with the highest degrees of negative regulation (Figure 2G). We also calculated the distribution of each gene enrolled in the Sankey network between IDH‐mutant and IDH‐wildtype status of glioma (Figure 2G).

### 2.4 Lower expression of *CTXN1* of histologically LGG associated with favorable prognosis

**FIGURE 3:**
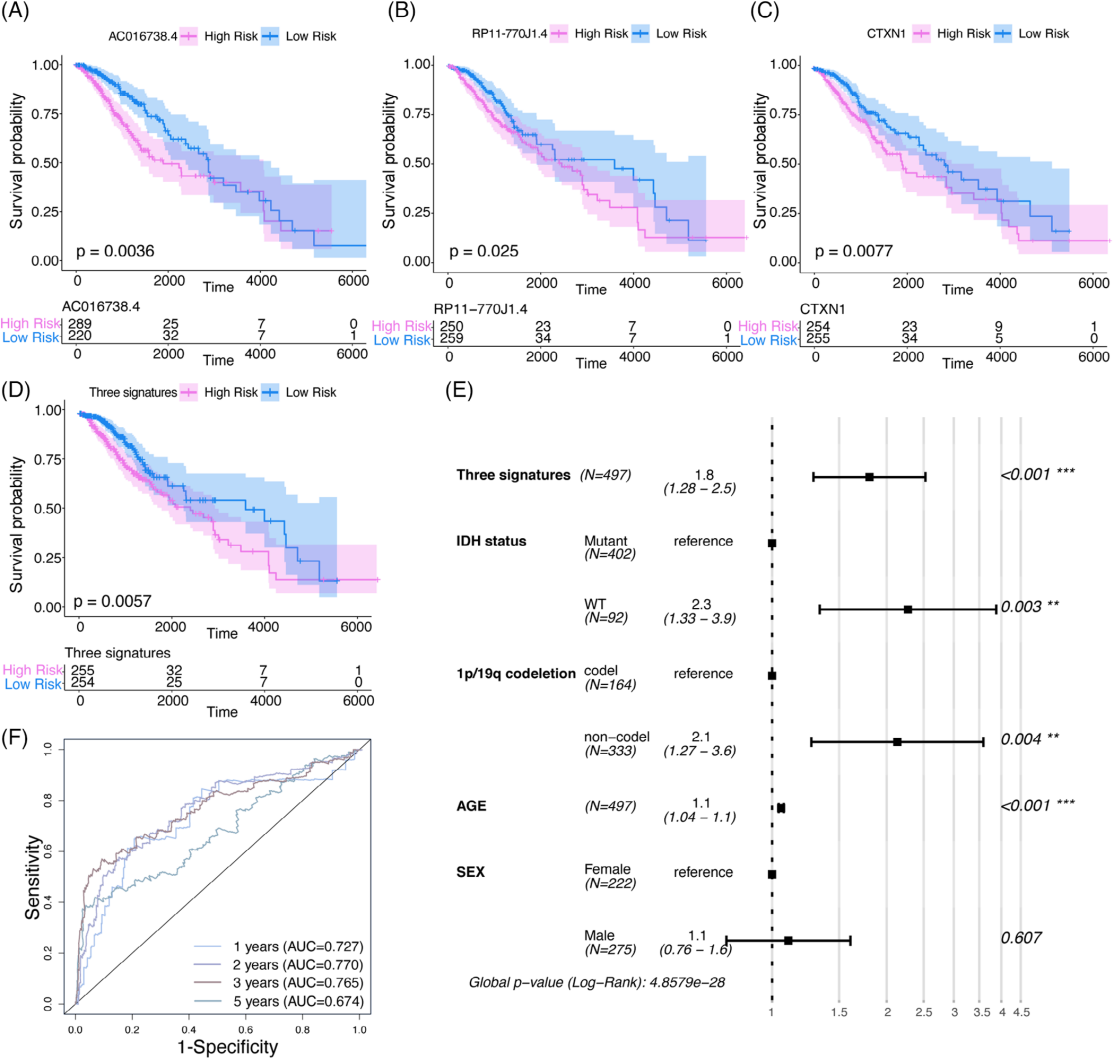
Identification of prognosis‐associated ceRNA regulatory axes. (A–C) Kaplan–Meier survival analysis of lncRNAs in (A and B) and mRNAs in (C) axes exhibiting a significant association with overall survival. Log‐rank tests were employed for *p*‐value calculations. (D) Kaplan–Meier survival analysis of a three‐gene signature. The signature was evaluated using three genes (lncRNA AC016738.4, lncRNA RP11‐770J1.4, CTXN1) based on Cox regression models. (E) Multivariate Cox regression analysis of the three‐gene signature was performed alongside several important prognostic factors in histologically LGG. (F) ROC curve analysis was conducted to assess the sensitivity and specificity of the signatures as survival markers (survival cut‐off time set at 1, 2, 3, 5 years). The area under the curve (AUC) value was calculated to indicate the predictive value of signatures in the models.

To examine the association between these genes and survival in histologically LGG, we conducted an OS analysis. Single‐factor Cox regressions were performed on each gene in the ceRNA network, identifying 38 DERNAs that were significantly associated with histologically LGG mortality (Table 1). Depending on the gene expression levels from 38 DERNAs, risk scores were calculated for each sample and divided patients into high‐risk and low‐risk groups. Low expression of one mRNA (*CTXN1*) and two lncRNAs (AC016738.4, RP11‐770J1.4) was associated with an optimal prognosis for histologically LGG (Figures 3A–C). We assigned patients to different risk groups depending on the expression levels of these three genes (Figure 3D), with individuals in the high‐risk groups having a worse prognosis. Then, we incorporated other acknowledged prognostic factors with this signature into COX regression model. The COX model showed that the histologically LGG signature was independent of several important clinical characteristics, such as gender, age, IDH status, and 1p/19q codeletion status (Figure 3E). We set survival cut‐off time into 1, 2, 3, 5 years, then used the receiver operating characteristic (ROC) curve to examine the sensitivity and specificity of signatures. The area under the curve (AUC) value is greater than 0.7 (Figure 3F), indicating our signatures have a good predictive value. In light of the above findings, two potential regulatory axes might be associated with prognosis in histologically LGG patients.

**TABLE 1:** Univariate COX analysis of DERNAs in histologically LGG.

Gene	*p* Value	Coefficient
RP11‐566J3.2	2.53E−15	0.01666324
CTD‐2201E18.3	1.18E−12	0.00332968
RP4‐580N22.2	5.16E−10	0.0381353
AC067960.1	8.29E−09	0.1542956
CTD‐2544H17.1	4.16E−07	0.00640501
AP000266.7	4.31E−07	0.0925276
AC062021.1	5.74E−07	−0.0023435
DBH‐AS1	1.71E−06	0.00354141
RP11‐379B8.1	6.44E−06	0.02343023
RP4‐694A7.2	9.55E−06	0.02163763
RP11‐1167A19.2	9.88E−06	0.15679365
CTD‐2353F22.1	1.60E−05	0.00906391
RP11‐174J11.1	5.54E−05	−0.0110072
AC016738.4	9.22E−05	0.00944215
ARPP21‐AS1	0.00038622	−0.032652
AP002954.4	0.000499	0.01825845
RP11‐770J1.4	0.00067232	−0.022132
RP11‐24F11.2	0.00092403	0.0052593
RP11‐360F5.1	0.00109689	0.02966772
RP11‐300A12.2	0.00116675	0.17503565
LINC00543	0.00147725	0.01969866
RP5‐1177M21.1	0.00149383	−0.0019526
DDIT4	0.00163302	−8.00E−05
LINC01036	0.00170024	0.06301162
GS1‐204I12.4	0.00182209	−0.2057623
AC114730.3	0.00209558	−0.0004442
AC083864.4	0.00294875	−0.0845822
hsa‐mir‐124‐2	0.00299134	−0.0606249
RP11‐148O21.4	0.00310488	−0.0376209
RP11‐620J15.2	0.00314238	0.0047514
AC007040.8	0.00375962	0.03868357
RP11‐395D3.1	0.00422349	0.00533761
CTD‐2116N24.1	0.0049474	−0.1546355
RP11‐1L9.1	0.00685558	0.11072719
SHANK2‐AS1	0.00691489	−0.0802921
LINC00336	0.00718404	−0.1012808
RP11‐238K6.1	0.00736418	−0.0326957
CTXN1	0.00771007	0.12269914

### 2.5 LncRNA RP11‐770J1.4 regulates CTXN1 expression through hsa‐miR‐124‐3p

**FIGURE 4:**
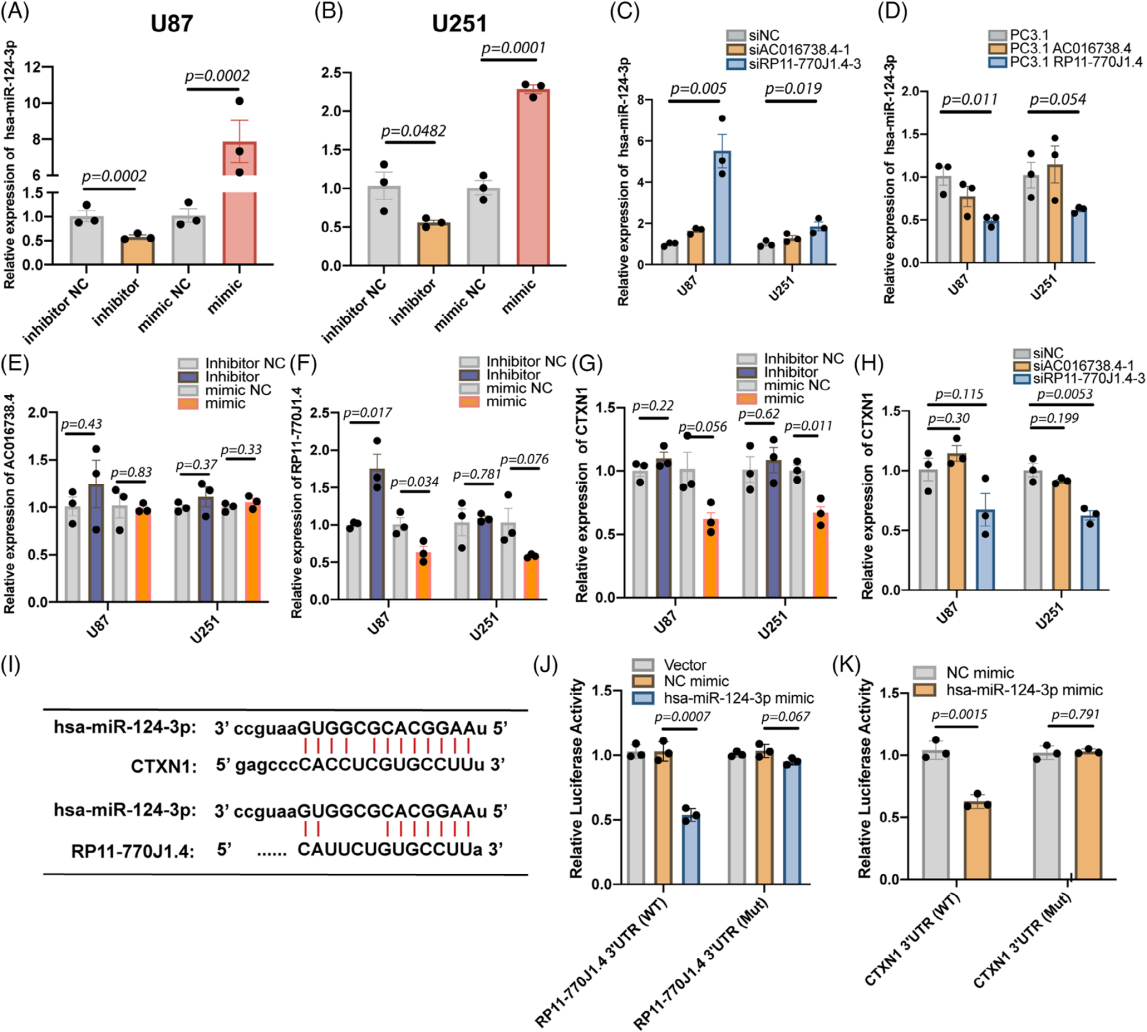
LncRNA RP11‐770J1.4 interacting with hsa‐miR‐124‐3p modulated the expression of CTXN1. (A and B) Quantification of hsa‐miR‐124‐3p expression in U87 and U251 cells was performed using qRT‐PCR after transfected miRNA inhibitors or mimics. All experiments were conducted in triplicate. (C) Evaluation of hsa‐miR‐124‐3p expression in U87 and U251 cells transfected with siRNA negative control (siNC), siAC016738.4, or siRP11‐770J1.4 using qRT‐PCR. (D) Examination of the effects of lncRNA AC016738.4 and lncRNA RP11‐770J1.4 overexpression on hsa‐miR‐124‐3p expression in U87 and U251 cells using qRT‐PCR. (E and F) Measurement of lncRNA AC016738.4 and lncRNA RP11‐770J1.4 expression in U87 and U251 cells post‐treatment with hsa‐miR‐124‐3p inhibitor, inhibitor NC, mimics, and mimic NC. (G) *CTXN1* expression was assessed by qRT‐PCR in U87 and U251 cells following transfected with hsa‐miR‐124‐3p inhibitor, inhibitor NC, mimic, and mimic NC. (H) *CTXN1* expression was measured by qRT‐PCR in U87 and U251 cells after transfection with siNC, siAC016738.4, and siRP11‐770J1.4. (I) Schematic illustration depicting the putative binding sites of hsa‐miR‐124‐3p with lncRNA RP11‐770J1.4 and the 3′UTR of *CTXN1*. (J and K) Relative luciferase activity of wildtype and mutant 3′UTR of lncRNA RP11‐770J1.4 and *CTXN1* co‐transfected with hsa‐miR‐124‐3p mimics after 48 h. NC: negative control; WT, wild type; MT, mutant type. One‐way ANOVA followed by Dunnett's test was used for multiple comparisons. Data are shown as the mean ± SEM. *p < 0.05, **p < 0.01.

To delineate the functional involvement of lncRNA RP11‐770J1.4–CTXN1 axis in tumor progression, validation experiments were conducted in two human GBM cell lines: U87 and U251. Hsa‐miR‐124‐3p is the matured sequence corresponding to miR‐124‐2 as per MiRbase. Thus, the miRNA inhibitors and mimics for hsa‐miR‐124‐3p were designed and validated (Figures 4A and B). Knockdown of lncRNA RP11‐770J1.4 exhibited a substantial reduction in *CTXN1* expression and a concurrent increase in hsa‐miR‐124‐3p expression, whereas the impact of lncRNA AC016738.4 siRNA was modest (Figures 4C and H). Conversely, overexpression of lncRNA RP11‐770J1.4 resulted in decreased expression of has‐miR‐124‐3p, while lncRNA AC016738.4 did not produce a comparable effect (Figure 4D). We also measured lncRNA RP11‐770J1.4, AC016738.4, and *CTXN1* expression through real‐time quantitative polymerase chain reaction (qRT‐PCR) after transfection with hsa‐miR‐124‐3p mimics or inhibitors. Overexpression of hsa‐miR‐124‐3p significantly reduced the expression levels of lncRNA RP11‐770J1.4 and *CTXN1* (Figures 4E–G). However, inhibition of hsa‐miR‐124‐3p did not exert a discernible effect on *CTXN1* expression levels (Figure 4G). We employed TargetScan and miRcode to predict hsa‐miR‐124‐3p binding sites on the 3′ untranslated region (3′UTR) of CTXN1 and lncRNA RP11‐770J1.4 (Figure 4I). Specific mutations were introduced to the binding sites on the 3′UTR of lncRNAs RP11‐770J1.4 and CTXN1 (Figure S2A). The full‐length wildtype or mutant lncRNA RP11‐770J1.4 was cloned into the pmirGLO vector, and luciferase reporter assays were performed. Co‐transfection with the pmirGLO‐RP11‐770J1.4‐WT vector and hsa‐miR‐124‐3p mimics significantly suppressed luciferase reporter activity in 293T cells. This repression was abolished by mutations in the hsa‐miR‐124‐3p binding seed region in lncRNA RP11‐770J1.4 (Figure 4J). Similarly, we confirmed that hsa‐miR‐124‐3p could target the 3′UTR of CTXN1 and that mutations in the 3′UTR of CTXN1 binding sites reversed the luciferase activity (Figure 4K). Next, we utilized U251 and U87 cell lines with RP11‐770J1.4 overexpression and siRNA knockdown (Figures S3A and B) to investigate the in vitro impact of lncRNA RP11‐770J1.4 on glioma cell proliferation through cell counting (Figures S3C–F). The results revealed that modulation of lncRNA RP11‐770J1.4 had a negligible impact on the cell growth rate and migration ability. We then established a subcutaneous tumor model in nude mice using these cell lines. The subcutaneous tumor volume in nude mice was measured after implantation and compared among groups. We found that knockdown or overexpression of lncRNA RP11‐770J1.4 had little influence on tumor growth in vivo (Figures S3G and H), suggesting that lncRNA RP11‐770J1.4 may have other roles in tumor microenvironment.

### 2.6 LncRNA RP11‐770J1.4 negatively associated with immune signatures in histologically LGGs

**FIGURE 5:**
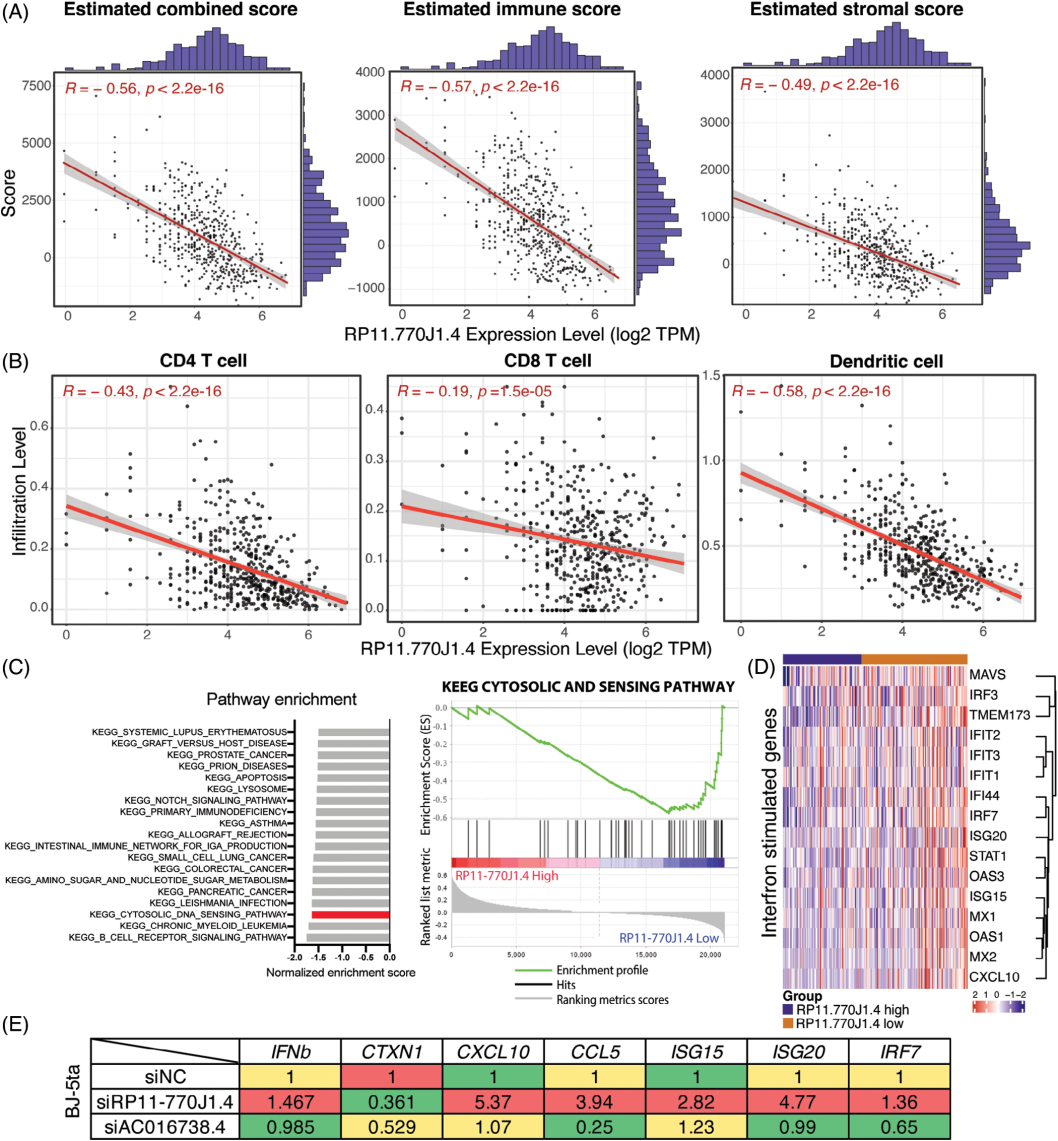
Expression of lncRNA RP11‐770J1.4 is inversely correlated with immune cell infiltration. (A) Pearson's correlation analysis was performed to evaluate the relationship between the ESTIMATE score and the expression level of lncRNA RP11‐770J1.4. The *y*‐axis indicates ESTIMATE scores while the x‐axis represents the expression levels of lncRNA RP11‐770J1.4. (B) Pearson's correlation analysis was conducted to assess the association between the expression level of lncRNA RP11‐770J1.4 and the TIMER‐estimated immune cell infiltration level. (C) On the left, a ranking plot displays significantly enriched pathways based on normalized enrichment scores. On the right, the enrichment of selected immune pathways was analyzed by GSEA for TCGA‐LGG patients in different lncRNA RP11‐770J1.4 expressed groups. (D) Heatmap of different lncRNA RP11‐770J1.4 expressed groups presented the expression levels of representative interferon‐stimulated genes. (E) qRT‐PCR of *IFNb*, *IRF7, CCL5*, *CXCL10*, *ISG15*, *ISG20*, and *CTXN1* in BJ‐5ta cells after transfected with siNC, siAC016738.4 and siRP11‐770J1.4. The median expression value was visualized by heatmap.

To investigate the role of immune cells infiltration in immune responses, we explored the expression of immune‐related lncRNAs in histologically LGG patients. We used ESTIMATE software^35^ to calculate the immune and stromal scores and found that low lncRNA RP11‐770J1.4 expression in patients was associated with higher immune‐infiltration scores (Figure 5A). This relationship is not observed in those with low expression of lncRNA AC016738.4 (Figures S2B and C). Next, software Tumor Immune Estimation Resource (TIMER)^36^ was employed to estimate infiltrating immune cell populations in each patient, including dendritic cells, CD4^+^, and CD8^+^ T cells. Spearman's rank correlation coefficient was applied to evaluate (|*R*| > 0.3 and *p* < 0.05). Immune cell infiltration was negatively correlated with lncRNA RP11‐770J1.4 expression (Figure 5B). We further used ImmLnc^37^ and found the association between lncRNA RP11‐770J1.4 with cytokines. KEGG pathway analysis in Table 2 also showed similar results. These suggested that lncRNA RP11‐770J1.4 may play a role in the adaptive immune response. Moreover, GSEA analysis revealed that the cytosolic DNA sensing pathway was highly enriched in the low expression of lncRNA RP11‐770J1.4 group (Figure 5C). Additionally, several interferon‐stimulated genes (ISGs), relating to the activation of type I IFN‐inflammatory response, were more highly expressed in cases with low expression of lncRNA RP11‐770J1.4 (Figure 5D). It has been reported that the stimulator of interferon genes (STING) mediates NF‐κB and IRF3‐dependent transcription of type I interferons and proinflammatory cytokines.^38^, ^39^ However, Low et al.^40^ showed that STING expression was epigenetically suppressed in both normal brain and glioma cells, but not in tumor‐associated immune cells or tumor stroma cells. Therefore, we used the fibroblast cell line BJ‐5ta to examine the functional effects of lncRNA RP11‐770J1.4 on the STING pathway response. We found that inhibition of lncRNA RP11‐770J1.4 increased ISGs in BJ‐5ta cells, indicating a higher type I IFN‐inflammatory response (Figure 5E). These findings suggest that lncRNA RP11‐770J1.4 functions as an immune‐negative regulator in histologically LGGs.

**TABLE 2:** GSEA revealed the correlation between RP11‐770J1.4 and cytokines.

lncRNA symbol	Immune pathway	*p* Value	ES	Score
RP11‐770J1.4	Antigen processing and presentation	0.0863	−0.3611	−0.8275
	Antimicrobials	0.1422	0.3309	0.7157
	BCR signaling pathway	0.2869	−0.3465	−0.4261
	Chemokines	0.2123	0.4038	0.5754
	Chemokine receptors	0.1964	−0.4251	−0.6071
	Cytokines	**0.0050** ^**^	0.4174	0.9900
	Cytokine receptors	0.8950	0.2542	−0.7900
	Interferons	0.8697	0.5598	−0.7395
	Interferon receptor	0.3464	−0.7534	−0.3072
	Interleukins	0.3647	0.4492	0.2706
	Interleukins receptor	0.9565	0.2600	−0.9130
	Natural killer cell cytotoxicity	0.8925	−0.2505	0.7850
	TCR signaling pathway	0.1032	−0.3734	−0.7937
	TGFb family member	0.9227	−0.2713	0.8454
	TGFb family member receptor	0.3356	−0.5216	−0.3289
	TNF family members	0.1993	0.6315	0.6014
	TNF family members receptors	1.0000	−0.1557	1.0000

* Shows statistical significance. **p<0.01

### 2.7 Low *CTXN1* expression corresponds to an enriched immune environment

**FIGURE 6:**
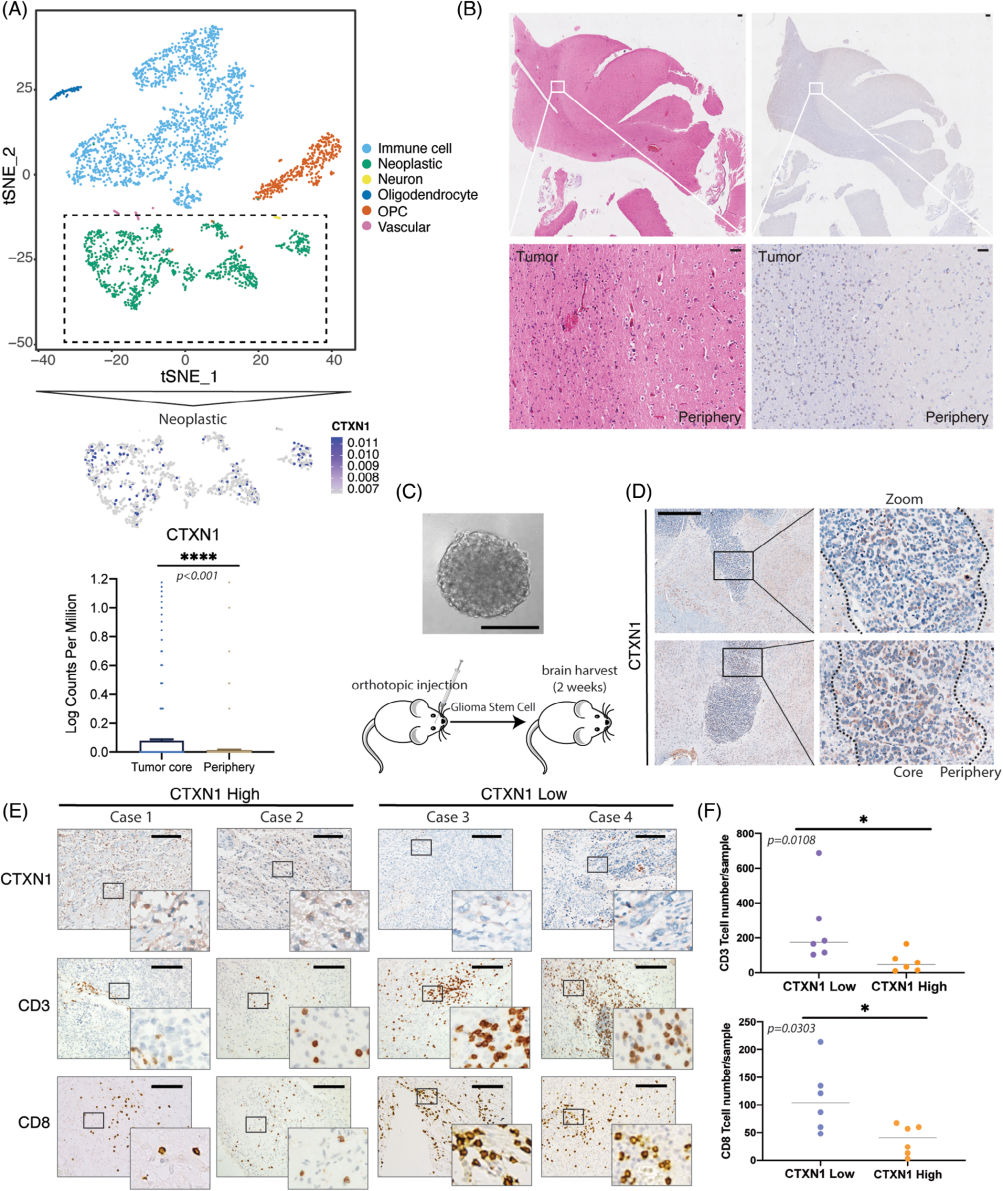
High expression of CTXN1 is predominantly localized in the tumor core and inversely correlated with CD8^+^ T cell infiltration. (A) scRNA‐seq data enrolled in this study were previously reported by Darmanis et al.^44^ The t‐SNE plot illustrates single‐cell clustering with annotated cell types, including oligodendrocyte progenitor cells (OPCs). Single gene expression depicted based on log_2_ counts per million was visualized using the DimPlot function of Seurat. The expression of *CTXN1* was found to be distributed in tumor core and peripheral tumors. (B) A representative case of paired IHC staining and H&E staining showed higher CTXN1 expression in tumor core in patient samples. (C) Upper panel: Sphere formation assays of MES28 cells; bottom panel: schematic diagram of GSC mouse model establishment. (D) Representative CTXN1 expression case of IHC staining in GSC mouse models. Mice were sacrificed after the implantation of GSC cells at 14 days. The peripheral tumor tissues refer to those around the border. (E) Representative cases of IHC staining depicting CD8, CD3, and CTXN1. (F) Twelve glioma cases were categorized into two groups based on the CTXN1 intensity. The total number of positive cells for CD3 and CD8 were counted in all slides at a magnification of 400×. Statistical analysis was performed using the Mann–Whitney test. *p < 0.05; ns, no significance. Data are presented as dots. (Scale bar, 200 μm in B, 100 μm in C, 50 μm in D, 40 μm in E.)

**FIGURE 7:**
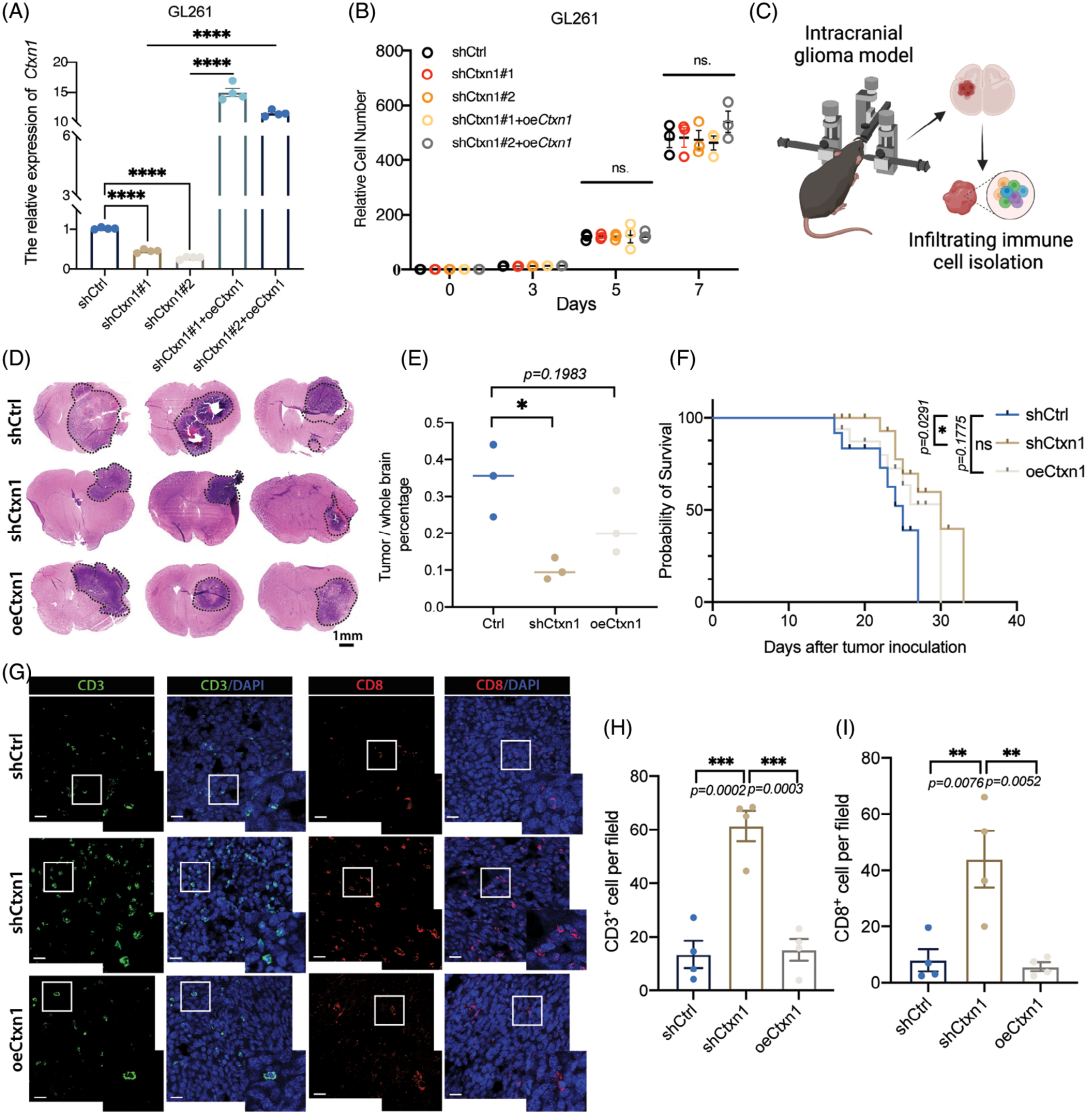
Knockdown of *Ctxn1* reduced tumor growth and improved survival via enhanced CD8^+^ T cell infiltration in vivo. (A) qPCR results of *Ctxn1* relative expression in shCtrl, shCtxn1, and oeCtxn1 GL261 cells. (B) cell count analysis of different groups. After the cells were diluted to 10,000 and planted into 12‐well plates, the cells were recounted at 3, 5, and 7 days, respectively. (C) Schematic diagram of mouse model and experiment. (D) Representative section with H&E staining from mice that orthotopically implanted shCtrl, shCtxn1, and oeCtxn1 GL261 cells. (E) Statistically results of the percentage of tumor size divide the whole brain section. (F) Survival analysis of asymptomatic mice implanted with either 5 × 10^4^ shCtrl or shCtxn1 GL261 cells (7 mice/group) was conducted. Log‐rank tests were employed for *p*‐value calculations. (G) Immunofluorescence staining of CD3 and CD8 revealed higher infiltration of CD3^+^ and CD8^+^ cells in the shCtxn1 group. Images of higher magnification are shown at the lower right. Shown are representative images from at least five cases with similar results. (H and I) Quantification of CD3^+^ and CD8^+^ cells in the area of three groups. Data are shown as the mean ± SEM. *p < 0.05, **p < 0.01, ***p < 0.001. (Scale bar: 1 mm in C, 20 μm in F.)

The tumor microenvironment consists of heterogeneous cell populations.^41^ Bulk RNA‐seq cannot capture the cellular diversity and interactions within the tumor.^42^, ^43^ Here, scRNA‐seq were used to investigate genes in different part of tumor.^44^ We analyzed 3505 cells and 20,725 genes into six clusters, as previously reported.^44^ The peripheral regions of tumors are recognized to exhibit a more pronounced immune signature. In subsets cluster of neoplasms, *CTXN1* demonstrated predominant expression in tumor cores (Figures 6A and B). We confirmed the spatial distribution of CTXN1 in both paired patient samples and GSC mouse models, revealing lower CTXN1 expression in the peripheral tumor region compared with the tumor cores (Figures 6B and D). Then, we also examined CD3^+^, CD8^+^ cells, and CTXN1 expression in glioma cases (Figure 6E). Higher numbers of CD3^+^ and CD8^+^ cells were observed in samples with low CTXN1 expression than in samples with high CTXN1 expression (Figures 6F and S4). To explore the functional role of *CTXN1* in glioma progression and immune response, we manipulated its expression in GL261 cells by shRNA‐mediated knockdown (shCtxn1) or overexpression (oeCtxn1) and validated the effects by qRT‐PCR (Figures 7A and S5A). We did not observe any significant difference in cell proliferation among each group (Figures 7B and S5B and C). Then, C57BL/6 mice were orthotopically implanted shCtrl, shCtxn1, and oeCtxn1 GL261 cells and symptom‐free survival was determined (Figures 7 and S5D and E). Randomly selected mice were harvested for HE staining. We found that shCtxn1 GL261 cells induced decreased tumor weight and prolonged survival than shCtrl or oeCtxn1 GL261 cells (Figures 7D–F). Moreover, shCtxn1 tumors showed increased infiltration of CD3^+^ and CD8^+^ T cells compared with shCtrl or oeCtxn1 tumors, as revealed by IF staining (Figures 7G–I) and flow cytometry (Figures S5F–H and S6).

## 3 DISCUSSION

The prognosis of histologically LGGs has improved a lot, benefiting from postoperative chemotherapy and radiotherapy.^45^ However, glioma treatment remains a clinical challenge. LncRNAs are involved in various biological processes, such as carcinogenesis, immune regulation, proliferation, invasion, and drug resistance.^46^ The lncRNA‒mRNA axis has emerged as a potential diagnostic and therapeutic target for gliomas.^47^ In this study, we obtained differentially expressed lncRNAs, miRNAs, and mRNAs from transcriptional profiles,^48^ and constructed a ceRNA network. LncRNA RP11‐770J1.4 could perform as a sponge for hsa‐mir‐124‐3p and regulate *CTXN1*. We also identified lncRNA RP11‐770J1.4 as an immune‐regulatory lncRNA. The Infiltration of CD8^+^ T cells is inversely correlated with CTXN1 expression in the tumor core rather than the periphery. Knocking down *Ctxn1* in GL261 cells prolonged survival and reduced tumor weight in mice‐bearing tumors, while increasing the infiltration of CD8^+^ T cells within tumors. These findings provide new insights into lncRNAs in histologically LGGs.

Because of the deficiency of the control brain in TCGA LGG cohort, it is difficult to construct a ceRNA network for histologically LGGs. To overcome this, we combined TCGA data with GTEx data for analysis. This allowed us to establish ceRNA networks to explore the regulatory mechanism of lncRNAs and to uncover the complex processes of glioma progression.^47^ We detected 703 DEmRNAs between histologically LGGs and normal brain tissues. The GO and KEGG functional enrichment analysis indicated that these DEmRNAs were mainly involved in functions related to the inflammatory response. These results suggested that immune response was tightly linked to the histologically LGGs.

Tumors exhibit aberrant expression of numerous miRNAs. In our survival analysis and ceRNA network, hsa‐miR‐124‐2 interacted with most DElncRNAs and DEmRNAs (connection degree = 41). Its expression is also associated with the prognosis of histologically LGG patients. Moreover, hsa‐miR‐124 is known to play a critical role in human tumorigenesis.^49^, ^50^, ^51^ The mature sequences of hsa‐miR‐124, hsa‐miR‐124‐3p, or hsa‐miR‐124‐5p, are derived from precursor sequences, which are miR‐124‐2, miR‐124‐1, and miR‐124‐3.^52^ hsa‐miR‐124 is often methylated in hepatocellular carcinoma and gastric cancer.^50^, ^53^ Abnormal hsa‐miR‐124 expression adversely impacts clinical outcomes, consistent with existing literature.^49^, ^54^, ^55^ Survival analysis of factors in the ceRNA network substantiates the prognostic significance of lncRNAs AC016738.4 and RP11‐770J1.4. Specifically, overexpression of lncRNA AC016738.4 is linked to diminished OS and relapse‐free survival times in esophageal cancer,^56^ establishing its potential as a biomarker for pancreatic ductal adenocarcinoma.^57^ Moreover, a phase II clinical trial involving patients with recurrent glioblastomas exhibiting EGFR amplification revealed that lncRNA RP11.770J1.4 is linked to the response to the combination of Depatux‐M and TMZ (HR 0.587, *p* = 0.050 and HR 0.322).^58^ Principal component analysis reduction of RNA profiles in CD4^+^TIM‐3^+^ T‐cell and CD4^+^TIM‐3^−^ T‐cell indicates dysregulation of lncRNA RP11.770J1.4 in colorectal cancer.^59^ Both lncRNAs hold promise as potential biomarkers for histologically LGGs.

LncRNAs are important regulators of immune response.^25^, ^37^, ^60^ The identification of lncRNA targets is a pivotal step in elucidating lncRNA functions. Within our ceRNA network, we pinpointed lncRNA RP11‐770J1.4, assessing its prognostic significance and investigating its involvement in immune pathways. Enrichment analysis unveiled a significant association between lncRNA RP11‐770J1.4 expression and cytokines, multifunctional polypeptides or glycoproteins with either pro‐ or anti‐inflammatory properties.^61^, ^62^ The activities of several cytokines are intricately regulated by the tumor immune microenvironment.^63^ We further delved into CTXN1, which is recognized for its role in mediating cortical neuron growth during development. ScRNA‐seq analysis, GSC models, and section‐paired patient samples collectively affirmed CTXN1 expression in gliomas. Interestingly, glioma samples with diminished CTXN1 expression exhibited elevated levels of CD3^+^ and CD8^+^ T cells. Notably, inhibition of lncRNA RP11‐770J1.4 prompted activation of cGAS–STING‐related ISGs, highlighting its role as an immune‐related lncRNA that modulates the tumor microenvironment. Further research is required to elucidate the detailed mechanism.

Despite these novel findings, our study has some limitations that should be considered. First, we compared all samples of TCGA‐LGG with normal tissue from normal frontal sites (frontal cortex BA9) in the brain, without subclasses of analysis. The differential gene comparisons resulting from these analyses are not representative of the complex variation present in all LGGs. In addition, the variant genes derived from our analysis are hardly representative of all the genes that play a role in the development of gliomas, and are more of an interpretation based on correlations. Third, the mechanism of lncRNA RP11‐770J1.4‐mediated regulation of CTXN1 and cGAS–STING pathway remains unclear and requires further investigation.

In summary, we constructed a ceRNA network using large‐scale TCGA and GTEx datasets and provided potential therapeutic targets. The elucidation of this ceRNA network enhances our understanding of lncRNA regulation, highlighting the RP11‐770J1.4–CTXN1 as a significant immune regulatory axis in gliomas.

## 4 METHODS

### 4.1 Data collection

We obtained patient data from TCGA database (https://xenabrowser.net/datapages/), including expression RNA profiles (511 samples), gene mutation characteristics and survival data (533 samples). We also downloaded GTEx database data titled normal brain tissues (Frontal Cortex BA9). The different types of RNA were retrieved and obtained from the ENSEMBL database (http://asia.ensembl.org/). GRCh38 was used as the reference genome for lncRNAs and mRNAs quantification.

### 4.2 Differential expression analysis

Using R (version 3.6.3), a differential expression analysis was performed to identify differentially expressed DEmRNAs,^64^ DElncRNAs, and DEmiRNAs by comparing TCGA‐LGG samples and GTEx samples. Corrected *p* values to the false discovery rate (FDR) were adjusted by Benjamini–Hochberg. All genes were screened in the conditions of |log_2_FC| ≥ 2 and FDR < 0.05 by edgeR package.

### 4.3 Functional pathway enrichment analysis

Enrichment analysis of GO^65^ and KEGG pathway^66^ of the DEmRNAs were conducted by clusterProfiler^67^ (R package) to elucidate the functional processes associated with DEmRNAs in histologically LGG. GO terms or KEEG pathways with FDR ≤ 0.05 were selected, and default parameters were used for the analysis. A KEGG pathway was considered as significant if *p* < 0.05.

### 4.4 ceRNA network construction

The DElncRNA‐targeted miRNAs were predicted using miRcode software.^68^ DElncRNA–DEmiRNA relationship pairs were considered as DElncRNAs that intersected with DEmiRNAs.

The target mRNAs of DEmiRNAs were predicted using TargetScan,^69^ miRDB,^70^ and miRTarBase.^71^ The final intersection results from these three tools was defined as miRNA–mRNA relationships. Finally, by intersecting the DEmRNAs and the predicted mRNAs, DEmiRNA–DEmRNA relationship pairs were obtained.

### 4.5 Survival analysis and ROC curve plot

Cox models were employed for individual genes or combinations of multiple genes utilizing the survival package in R. Genes reaching statistical significance (*p* ≤ 0.01) underwent multifactor survival analysis. Based on expression values and coefficients derived from the Cox regression, subjects were stratified into high‐ or low‐risk groups. Kaplan–Meier survival curves were utilized to visualize survival outcomes. Time‐dependent ROC curves were constructed using the survival ROC package in R, with cut‐off times set at 1, 2, 3, and 5 years. The AUC was determined through the Kaplan–Meier method.

### 4.6 Functional prediction of DEmRNAs

In the context of differentially expressed mRNAs, functional predictions were made using GSEA on the mRNAs within the network. This analysis was conducted to deduce the biological functions associated with the identified mRNAs. Based on the median value of mRNA expression, samples were classified into two groups (high expression vs. low expression). The c2.cp.kegg.v6.1.symbols.gmt, sourced from the Molecular Signatures Database (MSigDB), served as the reference gene set. Statistical significance for a gene set was determined at *p* < 0.05.

### 4.7 Cell culture and transfection

The Cell Bank of Institute of Biochemistry and Cell Biology of the Chinese Academy of Sciences (Shanghai, China) provided the glioma cell lines, U251 and U87‐MG. GL261 cells were gifts from Liufu Deng (Shanghai Jiao Tong University School of Medicine). BJ‐5ta cells were gifts from Qi Chen (Fujian Normal University). Dulbecco's modified Eagle medium (Gibco BRL, USA) containing 100 mg/mL streptomycin (Gibco BRL, USA) and 10% fetal bovine serum (Gibco BRL, USA) was used to culture the cells at 37°C with 5% CO_2_. Jeremy. N. Rich, University of Pittsburgh gifted patient‐derived primary glioblastoma stem cells: MES28. Neurobasal medium with B27 (without vitamin A; Invitrogen), 20 ng/mL epidermal growth factor (EGF), and 20 ng/mL basic fibroblast growth factor (FGF) were used to maintain GSCs. LipofectamineTM Transfection Reagent (Invitrogen) was used to transfect the cells with siRNAs, miRNAs, or plasmids for transient transfection according to the manufacturer's instructions.

### 4.8 RNA extraction and qRT‐PCR

The EZ‐press RNA purification kit (B0004D; EZBioscience, USA) was utilized to extract RNA, and a UV spectrophotometer was used to quantify it. gDNA remover was mixed with RNA and reverse transcribed into cDNA and amplified by EZ‐press Cell to cDNA Kit (B0003; EZBioscience, USA). A QuantStudio 6 Real‐Time PCR system (Applied Biosystems, Foster City, CA, USA) was used to perform qPCR using SYBR Green qPCR Master Mix (Vazyme Biotech, Nanjing, China). Primers were used and listed in Table S4. Three repetitions were done for all qPCR.

### 4.9 miRNA mimic/miRNA inhibitors/siRNA design and dual‐luciferase activity assay

RiboBio (Shanghai, China) designed and synthesized siRNA/miRNA mimic/miRNA inhibitors. Table S4 lists the detailed sequence information. Figures S3A and B validated the effects of siRNA used. Mutant and wild‐type 3′UTR sequences of the target gene were cloned into the pmirGLO dual‐luciferase reporter vector (Promega). LipofectamineTM reagent (Invitrogen) was used to transfect the NC/wild/mutant reporter vector and hsa‐miR‐124‐3p agomir into H293T cells. The dual‐Glo Luciferase Assay System (Promega) was used to detect the luciferase activities after 48h transfection of reporter plasmids in 293T.

### 4.10 Single‐cell data analysis

The scRNA‐seq data retrieved from the Gene Expression Omnibus database (GEO accession number: GSE84465) consisted of 3589 cells obtained from four human glioma samples.^44^ The dataset encompassed 2343 cells in tumor cores and 1246 cells in peripheral tumor regions, sequenced and generated with an Illumina NextSeq 500 platform. The entire set of 3589 cells underwent comprehensive analysis utilizing the Seurat package in R version 4.0.3. Rigorous quality control measures led to the exclusion of 84 cells deemed to be of low quality adhering to the following criteria: (1) exclusion of genes detected in fewer than three cells, (2) exclusion of cells with fewer than 50 identified genes, and (3) exclusion of cells with ≥5% mitochondria‐expressed genes. Visualization was achieved using t‐distributed stochastic neighbor embedding (t‐SNE). t‐SNE clustering by tumor region and presentation of marker genes for each cluster are provided in Figure S7. Cell types were annotated based on a previous report,^44^ and the resulting information is summarized in Table S5.

### 4.11 GSEA, correlation, ESTIMATE, and TIMER analysis

For functional analyses, GSEA identified pathways associated with different expression groups of lncRNA RP11‐770J1.4 using a glioma bulk RNA sequencing dataset.^72^, ^73^ The Benjamini–Hochberg FDR correction was applied to adjust *p* values, with an FDR ≤ 0.05 considered statistically significant. Single‐sample Gene Set Enrichment Analysis (ssGSEA) was performed using the R package GSVA. Pearson's correlation analysis was applied to assess the correlation between immune cell‐enriched scores. Visualization of the results was performed utilizing ggplot2, ggpubr, and ggExtra packages. Significance was determined by a *p* value < 0.05 and a correlation coefficient > 0.3 (absolute value). Additionally, the ESTIMATE analysis was carried out in R according to established protocols,^35^ and the code is available upon reasonable request. TIMER analysis was performed using the web version of the software (http://timer.cistrome.org/).

### 4.12 GSC model and patient samples

As previously described,^74^ GSCs were isolated from surgical specimens and subjected to functional verification through sphere formation and in vivo tumor formation assays. To support these functional assays, formalin‐fixed paraffin‐embedded (FFPE) specimens were procured from patients diagnosed with primary brain tumors. These patients underwent tumor resection at the Department of Neurosurgery, Huashan Hospital affiliated to Fudan University, between 2010 and 2013, and provided tumor tissue sections.

### 4.13 Animal experiments

All animal studies strictly adhered to approved protocols by the Ethics Committee of the Shanghai Medical School (202311017Z). A stereotaxic head frame, in conjunction with an anesthesia mask (stereotaxis for mouse, 68055 Adaptor), was employed to stabilize the mice during procedures. Anesthesia was induced using a 2% isoflurane–oxygen mixture. GL261 cells, suspended in 2 μL PBS, were stereotactically injected into the right striatum of 6−8 week‐old female C57BL/6J mice, with the specified cell quantity. The position is mapped as 2 mm posterior from bregma and 2 mm lateral from the coronal suture. For the GSC mice model, MES28 cells were stereotactically implanted into the craniums of nude mice (NSG, Shanghai Model Organisms), with 5 × 10^4^ cells per implantation. Mice were harvested for subsequent experiments at specific time points and were euthanized upon reaching veterinary endpoint symptoms (15% weight loss and/or exhibiting other clinical signs).

### 4.14 Immunohistochemistry staining

PBS and 4% paraformaldehyde perfused and fixed mouse brains for FFPE sections (4 μm), followed by deparaffinization and rehydration. Immunohistochemical staining was performed as described previously.^75^, ^76^ In brief, endogenous peroxidase was removed by 3% H_2_O_2_, and citrate buffer retrieved the antigens. For blocking, 5% normal sheep serum was employed, and primary antibodies (CD3: 1:100, R &D Systems MAB4841; CD8:1:50, R &D Systems MAB116; CTXN1: 1:100, ab121510) were incubated at 4°C overnight. Following three washes in TBS, corresponding secondary antibodies were incubated for 1 hour at 37°C. Visualization was facilitated by the Liquid DAB + substrate chromogen system (Dako, USA), and slides were counterstained with hematoxylin. Human tissue usage were sanctioned by the Ethics Committee of Shanghai Medical School Fudan University. The execution of all procedures strictly adhered to the approved guidelines (2019‐C014).

### 4.15 Tumor‐infiltrating cell flow cytometry

Following tumor trituration, a 40 μm cell strainer was employed to generate a single‐cell suspension. Erythrocytes were eliminated using lysis buffer for 5 minutes (Beyotime; C3702). Tumor‐infiltrating lymphocytes were enriched and filtered through density gradient centrifugation with isotonic 52% Percoll (Sigma; P4937) for 20 min at 1500 or 2000 rpm without brake. The resultant single‐cell suspensions were recounted and subsequently incubated with anti‐CD16/32 for 10 min at 4°C, followed by fluorescent antibody staining (1:200) for 30 min at 4°C. The antibodies used in this process were sourced from BioLegend and BD, including Horizon™ Fixable Viability Stain 780 (FVS780), CD3 (145‐2C11), CD4 (GK1.5), CD45 (30‐F11), and CD8 (53‐6.7). T‐cell populations were identified based on surface marker expression (CD45^+^ CD3^+^ CD4^+^ or CD8^+^).

### 4.16 Cell proliferation assay and wound healing

For the cell proliferation assay, 10,000 cells from different groups were seeded per well in 12‐well plates and continued incubation for 3 and 5 days. Cells were detached and counted at each time point. Ratio to the 10,000 or the absolute number of cells was compared at each time point. For the wound healing assay, after appropriate cells were incubated in 6‐well plates, a scratch in the monolayer was created by a sterile pipette tip. We washed the wells with PBS to remove any detached cells and debris. We captured images of the scratch area at 0 and 24 hours. The width of the gap at several points along the scratch was measured using ImageJ software. We calculated the wound healing rate as the percentage of gap closure between 0 and 24 hours.

### 4.17 Statistical analysis

Data were analyzed using R programming and commercially available software (SPSS 22.0). Differential expression analysis between tumor core and peripheral regions was performed using limma package (version 3.44.3). Gene sets from MSigDB with adjusted *p* value < 0.05 and normalized enrichment score (NES) > 0 or < 0 were considered as significantly enriched. Chi‐square tests were performed for categorical data, and two‐tailed *t*‐tests were applied for normally distributed variables. Group comparisons were assessed by one‐way ANOVA. Kaplan–Meier survival curves were plotted using survfit function and log‐rank test was used to compare the survival difference. The COX model was used to identify prognostic risk factors and to conduct causal mediation analyses of survival outcomes. Mann–Whitney *U* tests were applied for nonparametric data. The significance level was set at *p* < 0.05.

## AUTHOR CONTRIBUTION

Mao Ying, Hui Yang, and Chen Liang conceived and designed the study. Qiyuan Zhuang and Yihan Hu conducted the bioinformatics analysis. Chaxian Liu and Qiyuan Zhuang carried out the experimental procedures. Statistical analyses were performed by Qiyuan Zhuang. IHC staining and pathological examination were conducted by Ying Liu. Qiyuan Zhuang drafted the manuscript. Mao Ying, Hui Yang, Chen Liang, and Qiyuan Zhuang revised the manuscript. All authors critically reviewed and approved the final manuscript.

## CONFLICT OF INTEREST STATEMENT

The authors declare no competing interests.

## ETHICS STATEMENT

Human tissue usage were sanctioned by the Ethics Committee of Shanghai Medical School Fudan University. The execution of all procedures strictly adhered to the approved guidelines (2019‐C014). All animal studies strictly adhered to approved protocols by the Ethics Committee of the Shanghai Medical School (202311017Z).

## Supporting information

Supporting Information

Supporting Information

Supporting Information

Supporting Information

Supporting Information

Supporting Information

## Data Availability

The datasets utilized in this study are accessible in TCGA and GTEx public repositories. All data pertinent to this study, whether generated or analyzed, are comprehensively presented in this manuscript and its supplementary information. For any additional inquiries or requests, interested parties are encouraged to contact the corresponding authors.
