# Supplementary material for: Identification of RP11‐770J1.4 as immune‐related lncRNA regulating the CTXN1–cGAS–STING axis in histologically lower‐grade glioma
Source: MedComm (2020). 2023 Dec 19;4(6):e458. doi: 10.1002/mco2.458 (PMC10728758; doi:10.1002/mco2.458)
Supplement: Supplementary file 1 — Supporting Information [file MCO2-4-e458-s004.docx]

**Supplementary File**

**Article title: Identification of LncRNA RP11-770J1.4 as Immune-Related lncRNA regulating the CTXN1-cGAS-STING axis in histologically lower-grade glioma**

**Author:** Qiyuan Zhuang^1,8#^, Chaxian Liu^1,8#^, Yihan Hu^6,8#^, Ying Liu^6^, Yingying Lyu^1^, Yuheng Liao^7^, Liang Chen^1,3^*, Hui Yang^1,2,3,4^*, Ying Mao^1,3,4^*

**Affiliations:**

^1^ Department of Neurosurgery, Huashan Hospital, Fudan University, Shanghai, P.R. China;

^2^ Institute for Translational Brain Research, Shanghai Medical College, Fudan University, Shanghai, P.R. China;

^3^ National Center for Neurological Disorders, Huashan Hospital, Fudan University, Shanghai, P.R. China;

^4^ State Key Laboratory of Medical Neurobiology and MOE Frontiers Center for Brain Science, Institute for Translational Brain Research, Institutes of Brain Science, Fudan University, Shanghai, P.R. China

^5^School of Life Sciences, Fudan University, Shanghai, P.R. China;

^6^ Department of Pathology, School of Basic Medical Sciences, Fudan University, Shanghai, China;

^7^ Key Laboratory of Medical Epigenetics and Metabolism and Molecular and Cell Biology Lab, Institute of Biomedical Sciences, Shanghai Medical College, Fudan University, Shanghai, P.R. China;

^8^These authors contributed equally to this work


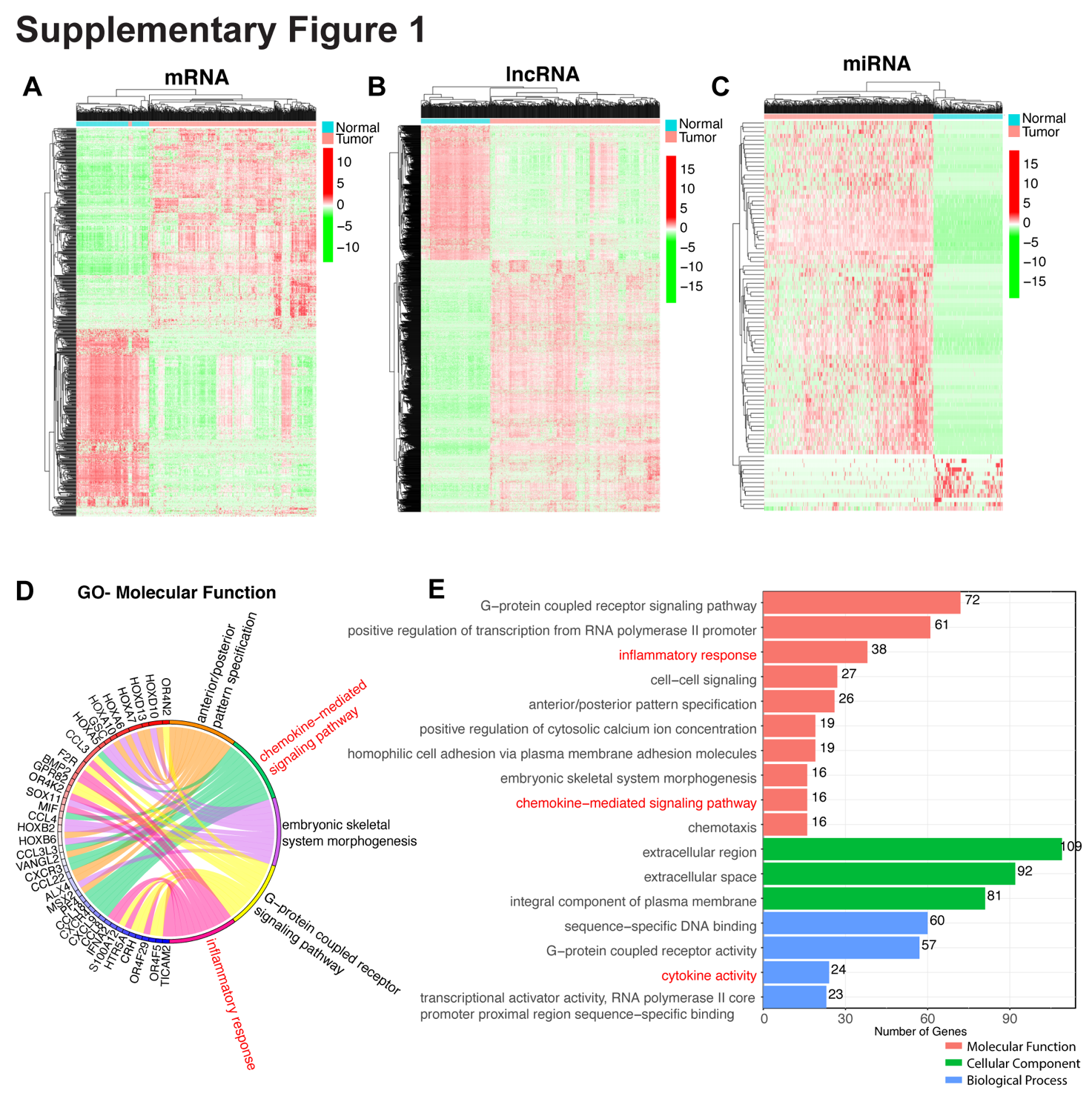


**Supplementary Figure 1. Extraction of DEGs in glioma.** (A-C) Heatmap plots of log_2_-adjusted fold changes of DElncRNAs (left panel), DEmiRNAs (middle panel) and DEmRNAs (right panel). The horizontal axis represents the individual samples, and the upper horizontal axis represents the sample clusters. DEGs and clusters are shown on the vertical axis. Red represents upregulated genes, while green represents downregulated genes. (D) Visualization of enriched MF (molecular function). The right half of the figure represents the functions or pathways, and the left half represents the genes. The colors of the genes change from red to blue according to the size of the log_2_-adjusted fold change. (E) Bar plot of functional GO enrichment of differentially expressed mRNAs. The top of the bar indicates the number of significantly enriched genes in each GO term.
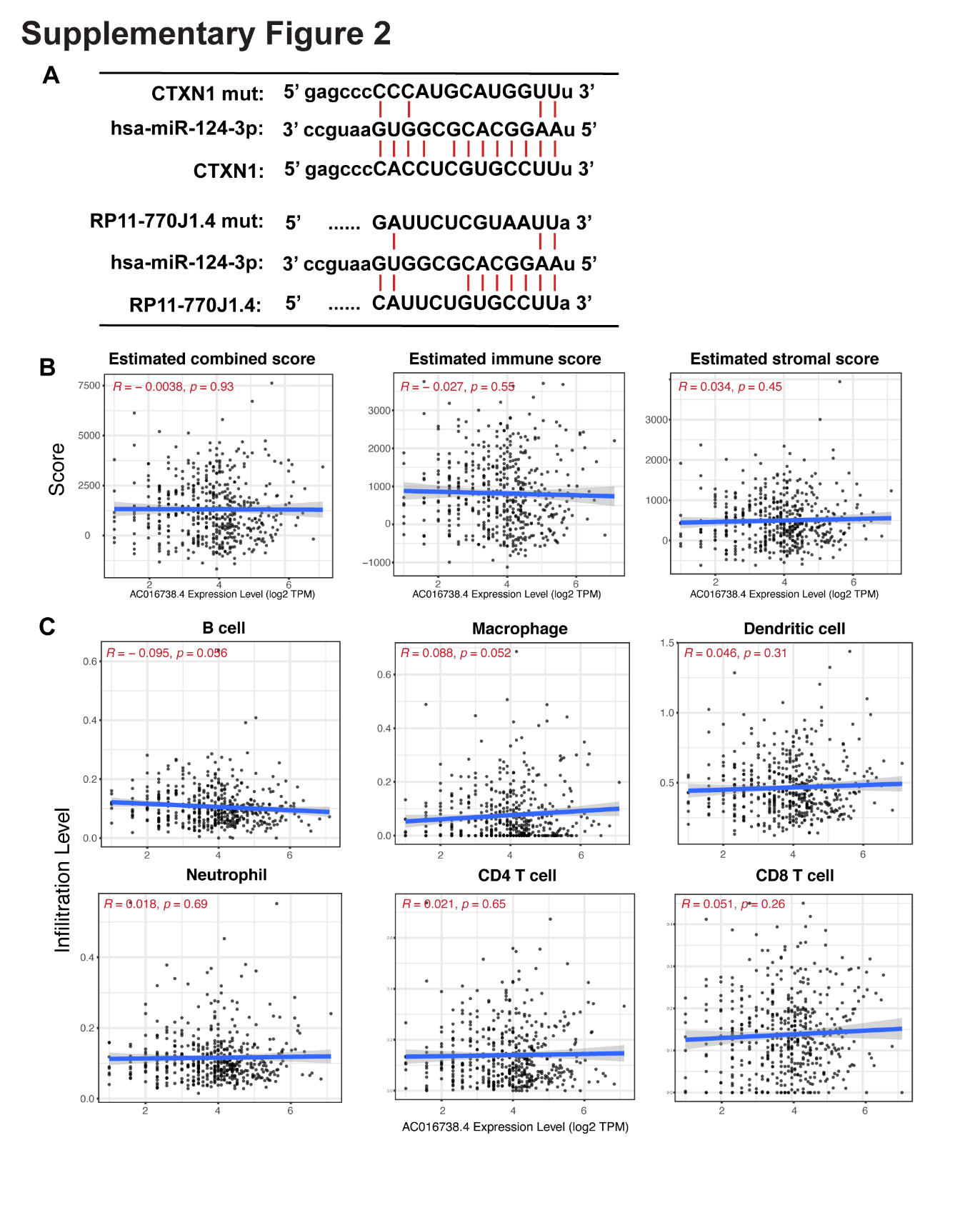
**Supplementary Figure 2. LncRNA RP11-770J1.4, but not lncRNA AC016738.4 is immune-related lncRNA.** (A) Schematic representation of the potential binding sites of hsa-miR-124-3p with lncRNA RP11-770J1.4 and the 3′UTR of *CTXN1*, and their corresponding mutation sites as well. (B) Regression analysis based on Pearson’s test between ESTIMATE score and the expression level of lncRNA AC016738.4. The horizontal axis denotes the expression levels of lncRNA AC016738.4, and the vertical axis represents ESTIMATE scores. (C) Regression analysis based on Pearson’s test between TIMER-estimated immune cell infiltration level and the expression level of lncRNA AC016738.4.


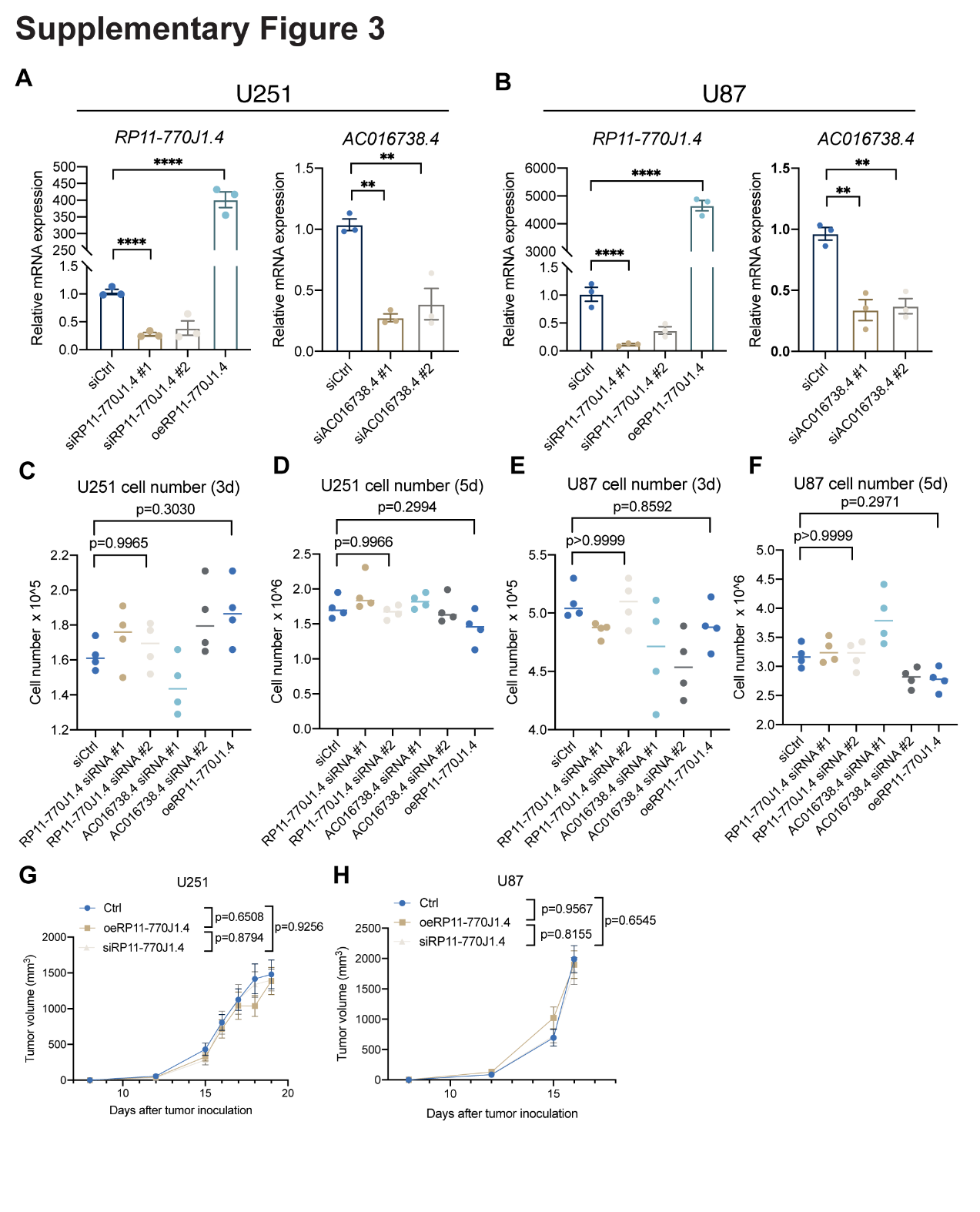


**Supplementary Figure 3. Effects of altered expression of lncRNA RP11-770J1.4 on glioma cells in vitro and in vivo.** (A-B) qRT-PCR results of siRNA or overexpression of lncRNA RP11-770J1.4 in U87 and U251. (C-F) cell count analysis of different groups. After the cells were diluted to 10,000 and planted into 12-well plates, the cells were recounted at 3 and 5 days, respectively. (G-H) Nude mice were implanted 5*10^6 cells of different groups of U87 and U251. Tumor volumes were measured regularly after a week. The size of the tumor is calculated by multiplying the height by half the bottom diameter squared.


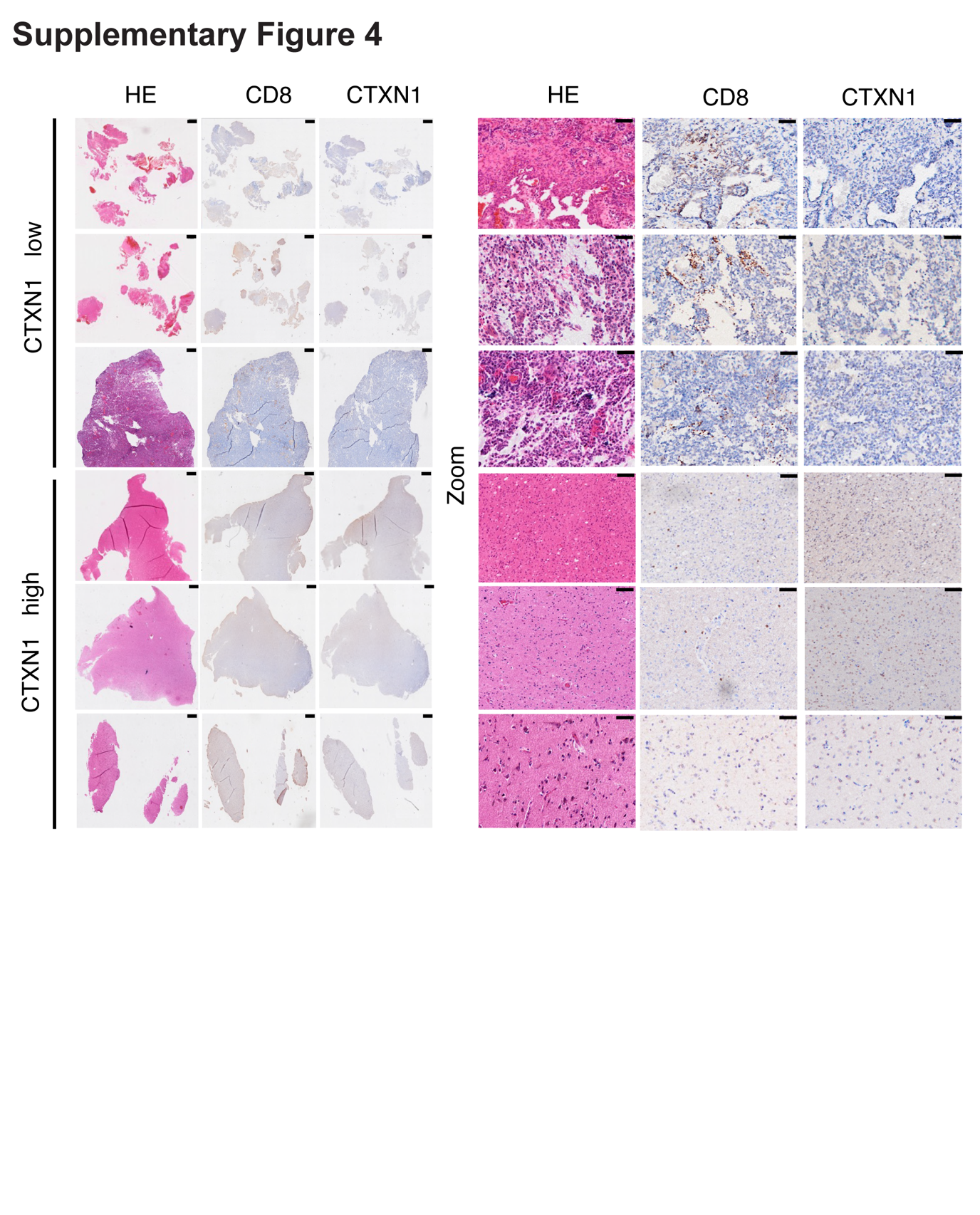


**Supplementary Figure 4. H&E staining of tissues and IHC staining of CD8^+^ T cells in different CTXN1 groups.** Scale bar: 2mm in the left, 400μm in the right.


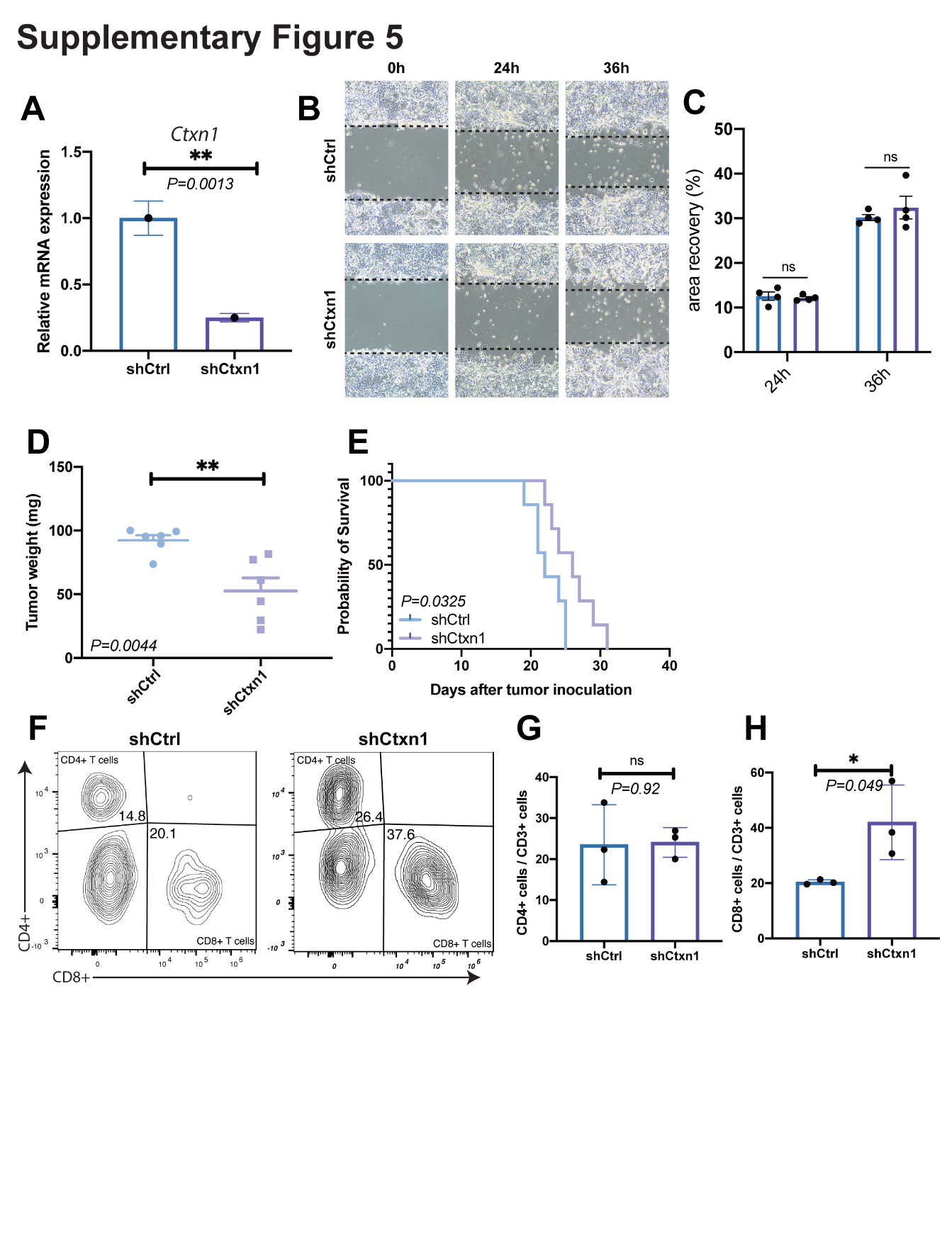


**Supplementary Figure 5. *Ctxn1*-KD in GL261 cells *in vivo* decreased tumor burden and prolonged survival via increased CD8^+^ T cell infiltration.** (A) Relative *Ctxn1* expression among shCtrl and shCtxn1 GL261 cells. Data are shown as the mean ± SD. ***P* <0.01*.* (B) Would-healing assay of shCtrl and shCtxn1 GL261 cells. (C) Statistical results of recovery rate of would-healing after 24h and 36h. (D) Harvested tumor weight comparison in the shCtrl and shCtxn1 groups 14 days after tumor implantation. Data are shown as the mean ± SD. ***P* <0.01*.* (E) Symptom-free survival of mice implanted with 5x10^4^ shCtrl or shCtxn1 GL261 cells. Survival curves represent accumulated data (7 mice/group) from two groups. (F) Mouse brain tumors were harvested 14 days after tumor implantation. The expression of CD4^+^ and CD8^+^ cell was examined by flow cytometry in tumor-infiltrating lymphocytes of the shCtrl and shCtxn1 groups. (G-H) Quantification of CD4^+^ and CD8^+^ cell expression in tumor-infiltrating lymphocytes between the shCtrl and shCtxn1 groups. **P* <0.05. Data are shown as dots (n=3). All the statistical results between two groups were analyzed by t-test.


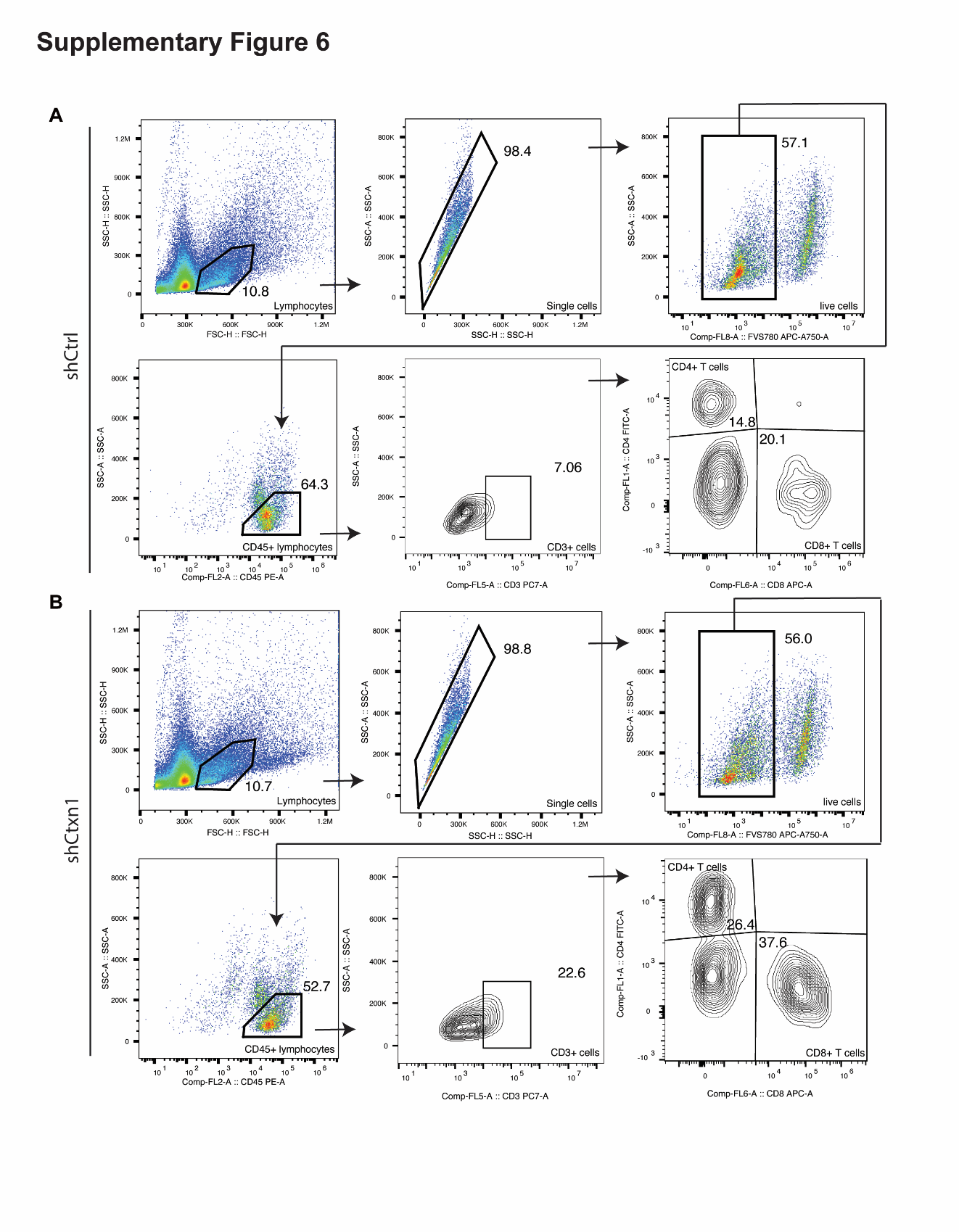


**Supplementary Figure 6. Gate strategy for examining CD4^+^ and CD8^+^ T cells among shCtrl and shCtxn1 groups.** (A-B) Flow cytometry analysis of CD4 and CD8 expression on tumor infiltrating cells from GL261 on day 21 after implantation.


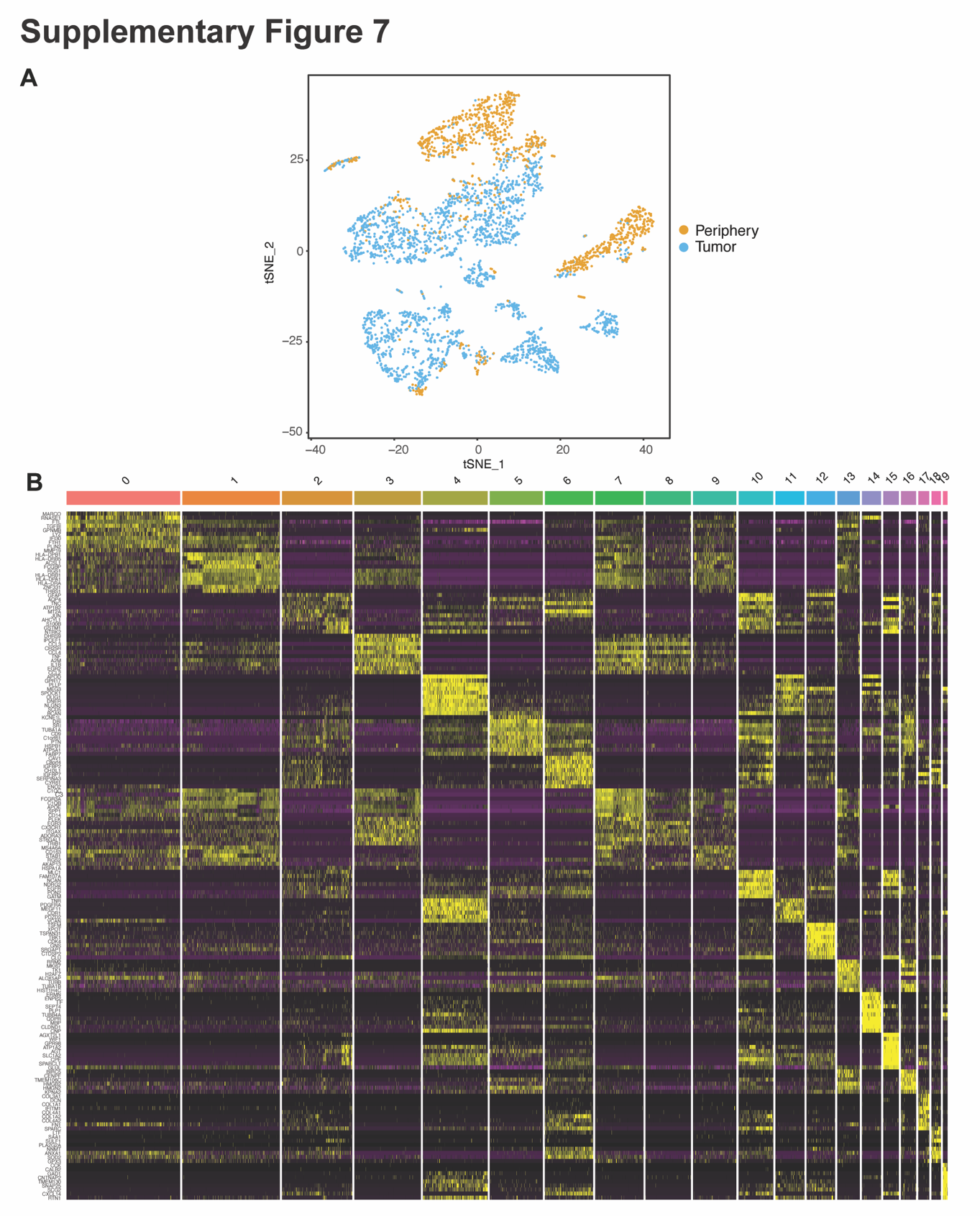


**Supplementary Figure 7. t-SNE plot clustered by tumor region and the marker gene of all clusters in scRNA-seq GBM datasets.** (A) GBM single cells were visualized by t-distributed stochastic neighbor embedding (t-SNE) analysis, and tumor cells derived from periphery and core were annotated by colors. (B) Heatmap of marker genes among clusters of all GBM cells.

Supplementary File 1. 1970 relationship pairs found by the intersection of DElncRNAs with DEmiRNAs.

Supplementary File 2. 2363 relationship pairs obtained by the intersection of the DEmiRNA–mRNA.

Supplementary File 3. 27 DEmRNAs were mapped among all pairs

Supplementary File 4. Detailed siRNA/ miRNA/ primer sequence information

Supplementary File 5. Detailed cell type annotated in figure S7.
