# Supplementary material for: Identification of RP11‐770J1.4 as immune‐related lncRNA regulating the CTXN1–cGAS–STING axis in histologically lower‐grade glioma
Source: MedComm (2020). 2023 Dec 19;4(6):e458. doi: 10.1002/mco2.458 (PMC10728758; doi:10.1002/mco2.458)
Supplement: Supplementary file 5 — Supporting Information [file MCO2-4-e458-s005.docx]

siRNA sequencing:

| Number | Name | Target |
| --- | --- | --- |
| siG2005180829216245 | si-h-ENST00000452364_001 | CTGGAACACTGTCCCTTTA |
| siG2005180829224201 | si-h-ENST00000532619_001 | CCACTGGCGAAACATTTGT |
| siN05815122147 | siRNA-negative control | TTCTCCGAACGTGTCACGT |

miRNA sequencing:

| Number | Name | Sense 5‘-3’ | Antisense 5‘-3’ |
| --- | --- | --- | --- |
| miR2180511012627 | micrOFFTM hsa-miR-124-3p inhibitor | UAAGGCACGCGGUGAAUGCCAA | UUGGCAUUCACCGCGUGCCUUA |
| miR1180418101457 | micrONTM hsa-miR-124-3p mimic | UUGGCAUUCACCGCGUGCCUUA | / |
| miR2N0000001 | micrOFFTM inhibitor Negative Control #22 | CAGUACUUUUGUGUAGUACAAA | / |
| miR1N0000001 | micrONTM mimic Negative Control #22 | UUUGUACUACACAAAAGUACUG | CAGUACUUUUGUGUAGUACAAA |

RT-qPCR primer：

| CTXN1-FP-01 | GTGTTCGCCTTCGTGCTCTG |
| --- | --- |
| CTXN1-RP-01 | AACGCGTAGTCGAACTGCC |
| CTXN1-FP-02 | GTGGCTCTGCTATTGTGCGT |
| CTXN1-RP-02 | CAAGGACGGGCTCAACACTC |
| has-124-3p-RT primer | GTCGTATCCAGTGCAGGGTCCGAGGTATTCGCACTGGATACGACTTGGCA |
| has-124-3p FP | CGTAAGGCACGCGGTGAA |
| has-124-3p RP | AGTGCAGGGTCCGAGGTATT |
| U6-FP | CTCGCTTCGGCAGCACA |
| U6-RP | AACGCTTCACGAATTTGCGT |
| ENST00000532619.1 -FP | TCACAACGACCCTCTCCCTC |
| ENST00000532619.1 -RP | AGTTCGCTGGAAGGTCATGC |
| ENST00000452364.1 -RP | GGAACTTGCGTCCCTTCTCT |
| ENST00000452364.1 -FP | GTTGCGTCTTAGTGACCCTGG |
